# NF-**κ**B represses retinoic acid receptor–mediated GPRC5A transactivation in lung epithelial cells to promote neoplasia

**DOI:** 10.1172/jci.insight.153976

**Published:** 2023-01-10

**Authors:** Hongyong Song, Xiaofeng Ye, Yueling Liao, Siwei Zhang, Dongliang Xu, Shuangshuang Zhong, Bo Jing, Tong Wang, Beibei Sun, Jianhua Xu, Wenzheng Guo, Kaimi Li, Min Hu, Yanbin Kuang, Jing Ling, Tuo Zhang, Yadi Wu, Jing Du, Feng Yao, Y. Eugene Chin, Qi Wang, Binhua P. Zhou, Jiong Deng

**Affiliations:** 1 Key Laboratory of Cell Differentiation and Apoptosis of the Ministry of Education and; 2 Shanghai Key Laboratory for Tumor Microenvironment and Inflammation, Shanghai Jiao Tong University School of Medicine, Shanghai, China.; 3 Department of Molecular and Cellular Biochemistry, Markey Cancer Center, University of Kentucky College of Medicine, Lexington, Kentucky, USA.; 4 Zhejiang Provincial Key Laboratory for Water Environment and Marine Biological Resources Protection, College of Life and Environmental Science, Wenzhou University, Wenzhou, China.; 5 Department of Pulmonary Medicine, Shanghai Chest Hospital, Shanghai Jiao Tong University, Shanghai, China.; 6 Medical Research Center, Binzhou Medical University Hospital, Binzhou, China.; 7 Peninsula Cancer Center, Binzhou Medical University, Yantai, China.; 8 Department of Respiratory Medicine, the Second Affiliated Hospital, Dalian Medical University, Dalian, China.

**Keywords:** Oncology, Cancer, Signal transduction, Tumor suppressors

## Abstract

Chronic inflammation is associated with lung tumorigenesis, in which NF-κB–mediated epigenetic regulation plays a critical role. Lung tumor suppressor G protein–coupled receptor, family C, member 5A (GPRC5A), is repressed in most non–small cell lung cancer (NSCLC); however, the mechanisms remain unclear. Here, we show that NF-κB acts as a transcriptional repressor in suppression of GPRC5A. NF-κB induced GPRC5A repression both in vitro and in vivo. Intriguingly, transactivation of NF-κB downstream targets was not required, but the transactivation domain of RelA/p65 was required for GPRC5A repression. NF-κB did not bind to any potential cis-element in the GPRC5A promoter. Instead, p65 was complexed with retinoic acid receptor α/β (RARα/β) and recruited to the RA response element site at the GPRC5A promoter, resulting in disrupted RNA polymerase II complexing and suppressed transcription. Notably, phosphorylation on serine 276 of p65 was required for interaction with RARα/β and repression of GPRC5A. Moreover, NF-κB–mediated epigenetic repression was through suppression of acetylated histone H3K9 (H3K9ac), but not DNA methylation of the CpG islands, at the GPRC5A promoter. Consistently, a histone deacetylase inhibitor, but not DNA methylation inhibitor, restored GPRC5A expression in NSCLC cells. Thus, NF-κB induces transcriptional repression of GPRC5A via a complex with RARα/β and mediates epigenetic repression via suppression of H3K9ac.

## Graphical abstract

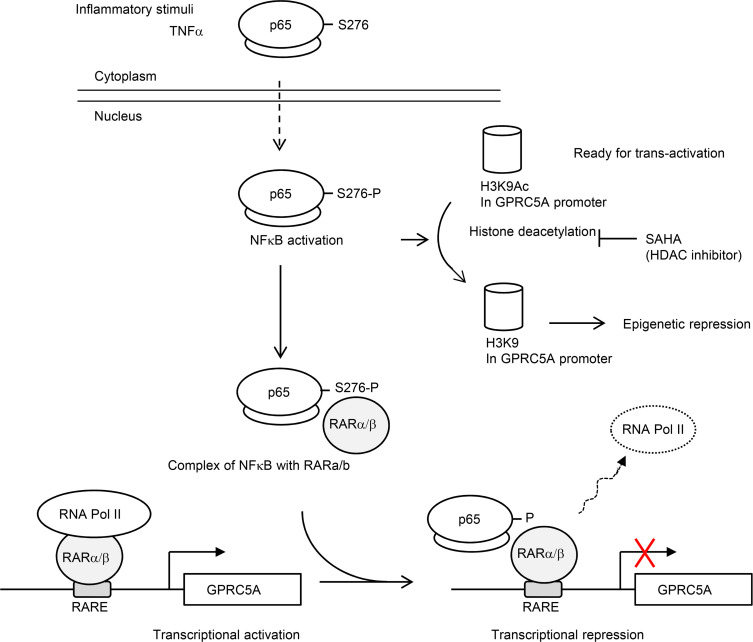

## Introduction

Tumorigenesis, a dedifferentiation process, is associated with chronic inflammation (1–3). However, the molecular mechanism that switches the expression profile from homeostasis to dedifferentiation remains elusive. Lung is an organ with exposure to various environmental insults, such as bacterial and viral infection, air pollution, and cigarette smoking. These risk factors trigger chronic inflammation, which releases reactive oxygen and nitrogen species, causing DNA damage and mutations (4). Proinflammatory cytokines from the microenvironment can activate the NF-κB signaling pathway, a pivotal transcriptional factor for induction of multiple downstream genes, in target cells. These downstream NF-κB targets include molecules involved in antiapoptosis, cell survival, proliferation, angiogenesis, and immune evasion. Up to now, nearly all NF-κB–mediated functions had been attributed to its transactivation activities, or activation of NF-κB downstream genes. However, little is known about the role of NF-κB in repression of the genes for homeostasis or differentiation.

G protein–coupled receptor, family C, member 5A (GPRC5A), was cloned as retinoic acid-inducible gene 1 (RAIG1) (5). GPRC5A is predominately expressed in lung epithelial cells (6), suggesting that it plays a differentiation role in maintaining homeostasis of lung epithelium. *Gprc5a*-knockout (-KO) mice have normal lung development, but develop spontaneous lung cancer in later life. Thus, *Gprc5a* is recognized as a lung tumor suppressor gene (6, 7). The biological functions of GPRC5A are linked to its roles in restraining EGFR signaling (8), inhibition of EGFR protein synthesis (9), inhibition of activated NF-κB and STAT3 signaling (10–12), and regulation of the MDM2/p53 pathway (13). Importantly, GPRC5A repression is found in human non–small cell lung cancer (NSCLC) samples and chronic obstructive pulmonary disease (COPD) tissues (6, 8, 14). Furthermore, GPRC5A repression is correlated with dedifferentiation grade of oral squamous cell carcinoma (15). These observations suggest that GPRC5A is a biomarker of differentiation in lung and oral epithelial cells, whereas repression of GPRC5A promotes dedifferentiation or a pro-oncogenic process for neoplasia. However, the underlying mechanism of GPRC5A repression remains elusive.

In this study, we showed that NF-κB acts as a transcription repressor to repress GPRC5A expression. We showed that NF-κB, via interaction with retinoic acid receptor (RAR), inhibits RAR-mediated transcription of GPRC5A, switching the gene expression from homeostasis to dedifferentiation in lung epithelial cells for neoplasia.

## Results

### Active NF-κB is strongly correlated with GPRC5A repression in human NSCLC and COPD samples.

**Figure 1:**
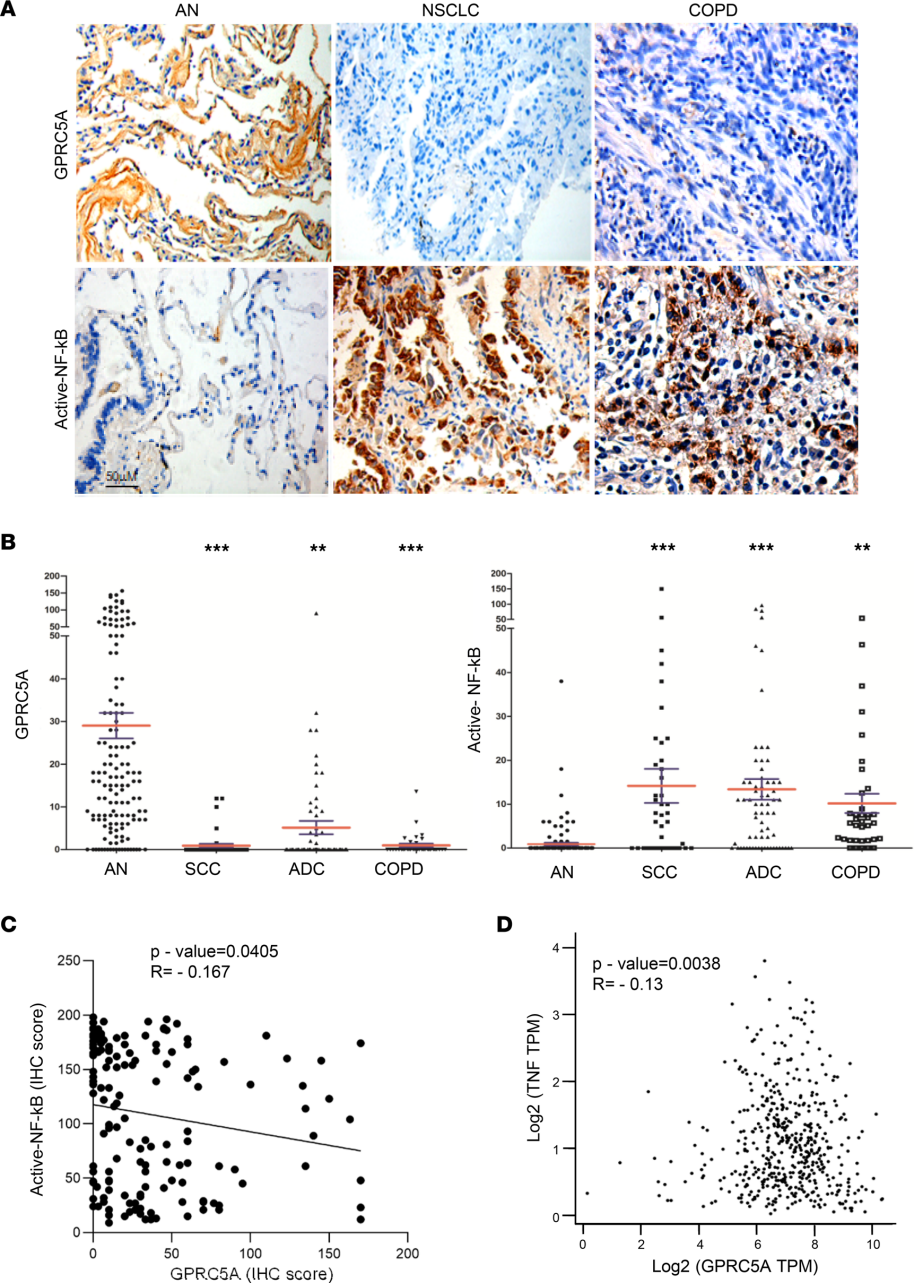
Active NF-κB is associated with GPRC5A repression in human NSCLC and COPD clinical samples. (**A**) Representative images of IHC staining for GPRC5A and activated NF-κB in human clinical samples. Scale bar: 50 μm. (**B**) Quantification of the IHC staining is represented as IHC scores of GPRC5A and activated NF-κB in human clinical samples; data are presented as the mean ± SD. (**C**) IHC scores of GPRC5A and activated NF-κB in human NSCLC clinical samples and AN tissues were analyzed by Pearson’s correlation. (**D**) Pearson’s correlation analysis of mRNA expression of TNF-α and GPRC5A in human LUAD clinical samples from TCGA database. Analyzed by 2-tailed Student’s *t* test; **P < 0.01; ***P < 0.001. AN, adjacent normal; SCC, squamous cell carcinoma; ADC, adenocarcinoma; TPM, transcripts per million.

GPRC5A is predominately expressed in lung epithelial cells, but its expression is often lost in lung cancer. To determine the relationship between NF-κB activation and GPRC5A expression, we measured the levels of GPRC5A expression and active p65 (nuclear p65) in samples via immunohistochemistry (IHC) staining. The results showed that GPRC5A expression was significantly repressed, whereas active p65 was greatly increased, in most of NSCLC, including squamous cell carcinoma (SCC) and adenocarcinoma (ADC), and all COPD tissues, compared with that in adjacent normal (AN) lung tissues (Figure 1, A and B). Pearson’s correlation analysis showed that GPRC5A protein level and active p65 were inversely correlated in normal lung, NSCLC, and COPD tissues (Figure 1C). Further analysis of The Cancer Genome Atlas (TCGA) database showed that the mRNA level of TNF-α, a key cytokine responsible for NF-κB activation, was inversely correlated with GPRC5A expression in lung adenocarcinoma (LUAD) (Figure 1D). Taken together, these results suggest that activated NF-κB is strongly correlated with GPRC5A repression.

### Inflammatory stimuli repress GPRC5A expression both in vitro and in vivo.

**Figure 2:**
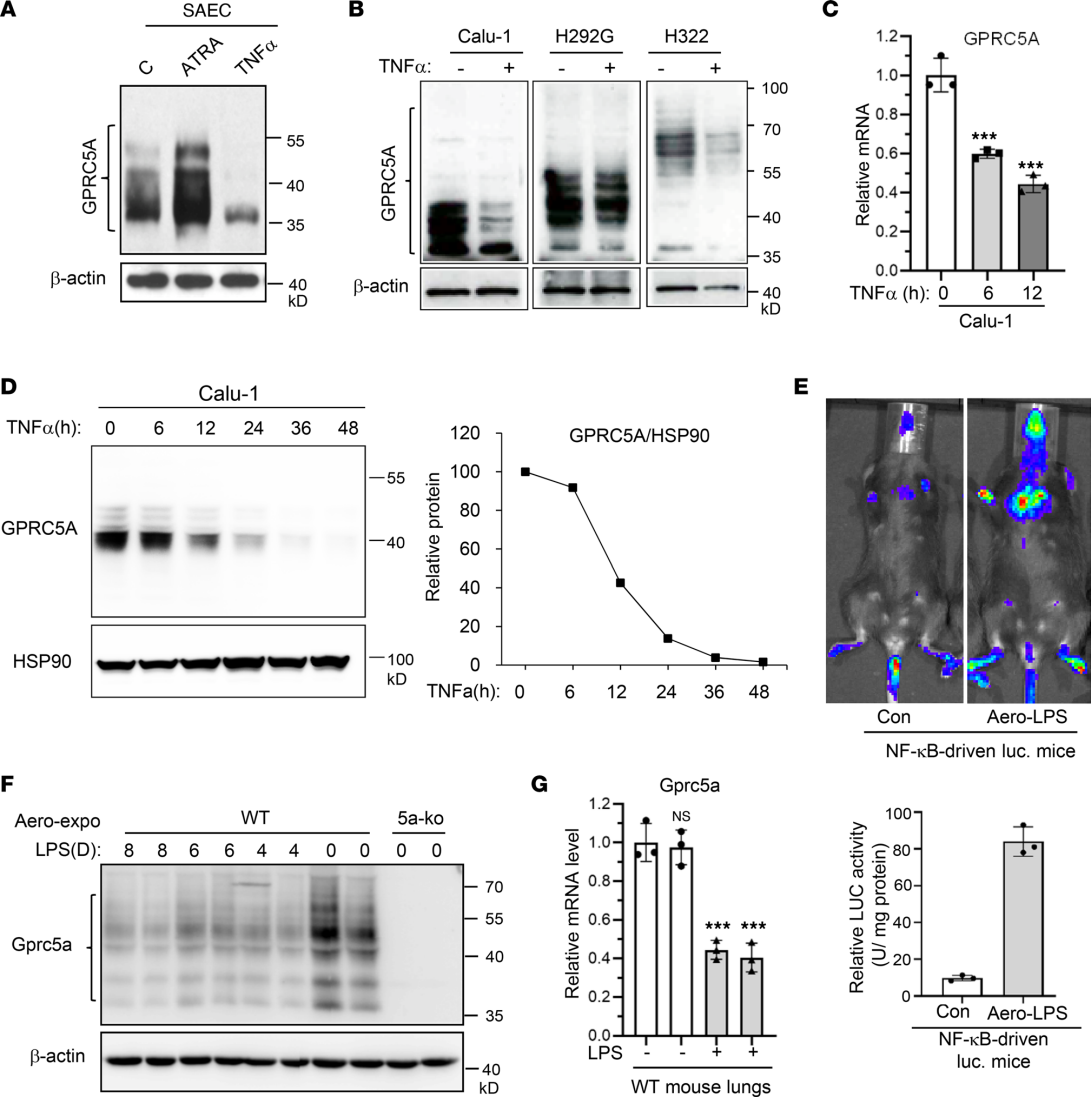
Inflammatory signaling inhibits GPRC5A both in vitro and in vivo. (**A**) Human small airway epithelial cells (SAECs) were treated with ATRA (1 μM), TNF-α (10 ng/mL), or cigarette smoke extract (CSE) (1 μM) for 24 hours, and the protein level of GPRC5A was analyzed by Western blotting. (**B**) Calu-1, H292G, and H322 cells were treated with TNF-α (10 ng/mL) for 24 hours, and the protein level of GPRC5A was determined by Western blotting. (**C** and **D**) Calu-1 cells were treated with TNF-α (10 ng/mL) for indicated times. The levels of mRNA (**C**) and protein (**D**) of GPRC5A were determined by quantitative PCR (qPCR) and Western blotting, respectively. Data are presented as the mean ± SD. (**E**) Representative data of luciferase activity of NF-κB–driven luciferase-transgenic mice treated with placebo or LPS (500 μg/5 mL) for 30 minutes through inhalation. (**F**) Mice were treated with LPS through inhalation for various days and the mouse lung tissue was analyzed for Gprc5a expression via Western blotting; data are presented as the mean ± SD. (**G**) Mice were treated with LPS through inhalation for 4 days, and lung tissue was collected; the mRNA level of Gprc5a was determined by qPCR. All data are presented as mean ± SD from 3 independent experiments with duplicates and analyzed by 2-tailed Student’s *t* test; ***P < 0.001.

To determine the role of inflammatory signaling on GPRC5A expression, we examined the effects of TNF-α in small airway epithelial cells (SAECs). Immunoblot assay showed that all-trans retinoic acid (ATRA) induced GPRC5A expression, demonstrating that GPRC5A is a retinoic acid (RA) target gene (5, 16, 17) (Figure 2A). Interestingly, treatment with TNF-α as well as CSE suppressed ATRA-induced GPRC5A expression. Similarly, TNF-α treatment inhibited GPRC5A expression in several NSCLC cell lines (Calu-1, H322, and H292G cells) (Figure 2B). Quantitative PCR (qPCR) analysis showed that the mRNA level of GPRC5A was significantly suppressed after 6 hours of TNF-α treatment (Figure 2C). In comparison, GPRC5A protein level started to decrease after 12 hours of TNF-α treatment in Calu-1 cells (Figure 2D). These results suggest that TNF-α represses GPRC5A at the transcriptional level.

Next, we examined the effect of inflammation on GPRC5A repression in vivo using an NF-κB–driven luciferase (NF-κB–luc) mouse model. After aero-exposure of these mice to LPS (aero-LPS) for 30 minutes, luciferin was injected intraperitoneally (i.p.) to visualize NF-κB activation. The images showed that aero-LPS exposure induced intensive bioluminescence in the lungs of these mice (Figure 2E, top); quantitation of relative bioluminescence intensity demonstrated NF-κB activation in these mice (Figure 2E, bottom). When lung tissues from these mice were analyzed for Gprc5a expression, immunoblot showed that Gprc5a was substantially suppressed following aero-LPS exposure for 4–8 days (Figure 2F). Consistently, qPCR analysis showed that Gprc5a mRNA was greatly suppressed in mouse lung tissues treated with aero-LPS for 4 days compared with untreated ones (Figure 2G), indicating that NF-κB–mediated repression of Gprc5a occurs at the transcription level. Taken together, inflammatory stimuli, TNF-α used in vitro or LPS used in vivo, significantly suppress GPRC5A expression at the transcriptional level both in vitro and in vivo.

### The transactivation domain of p65 is essential for NF-κB–mediated GPRC5A repression.

**Figure 3:**
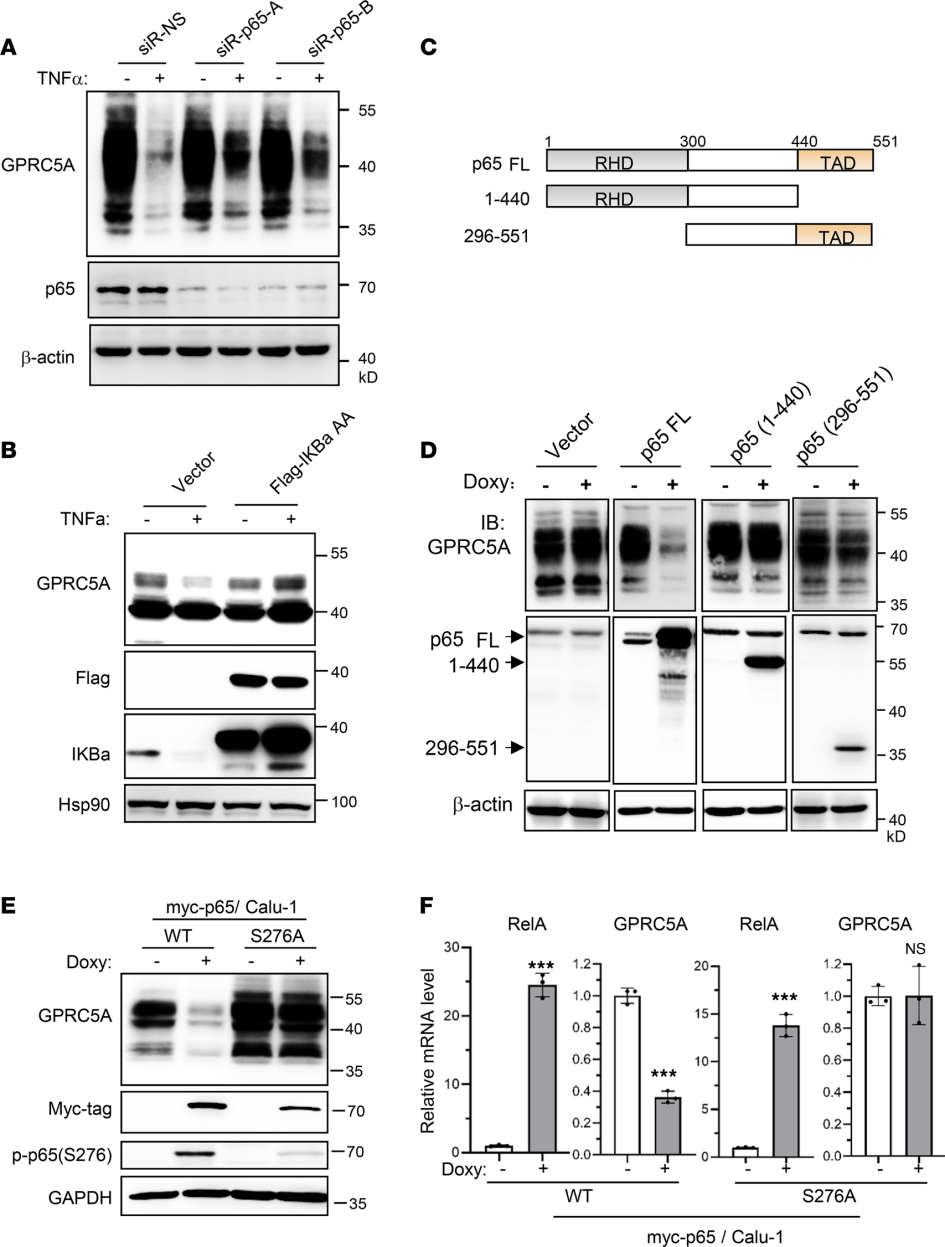
The transcription activation domain of NF-κB is required for GPRC5A repression. (**A**) Small interfering RNA (siRNA) targeting p65 and scramble control siRNA were transfected to Calu-1 cells, treated with or without TNF-α. The protein levels of GPRC5A and p65 were determined by Western blotting. (**B**) Calu-1 cells were transfected with plasmid overexpressing IκBα-AA mutant (S32A, S36A) or vector control, then treated with or without TNF-α; GPRC5A and IκBα protein levels were determined with specific antibodies through Western blotting. (**C**) Schematic representation of WT and truncation mutant of RelA/p65. (**D**) Calu-1 transfectants harboring vector control or inducible expression of FL and truncated p65 were established; cells were treated with doxycycline (300 ng/mL) for 24 hours, and GPRC5A protein levels were analyzed by Western blotting. These blots were run in separate gels performed in parallel with equal loading (please see uncropped/unedited gels in the supplement). (**E** and **F**) Calu-1 cells with inducible expression of WT p65 and serine 276A mutant were treated with doxycycline (300 ng/mL) for 24 hours; GPRC5A protein and mRNA levels were determined by Western blotting (**E**) and qPCR (**F**), respectively. Data are presented as mean ± SD from 3 independent experiments with duplicates and analyzed by 2-tailed Student’s *t* test, ***P < 0.001.

NF-κB is a major intracellular mediator of inflammatory signaling elicited from TNF-α. To determine whether NF-κB is the mediator responsible for TNF-α–induced GPRC5A repression, we examined the role of TNF-α in cancer cells treated with either RelA/p65 (subunit of NF-κB) knockdown by small interfering RNA (siRNA) or overexpression of dominant-negative inhibitor IκBα S32/36A mutant (IκBα-AA). The immunoblot assay showed that p65 knockdown abolished TNF-α–induced GPRC5A repression in Calu-1 cells (Figure 3A). Similarly, overexpression of IκBα-AA blocked TNF-α–induced GPRC5A repression (Figure 3B). This suggests that TNF-α–induced repression of GPRC5A is through NF-κB.

To define the functional domain of p65 that is responsible for the repression, we constructed inducible lentiviral expression constructs of full-length (FL) p65, or its mutant with deleted transactivation domain (TAD, 1–440 residues) or Rel homology domain (RHD, 296–551 residues) (Figure 3C), and established stable clones in Calu-1 cells. Immunoblot assay showed that doxycycline-induced expression of p65 (FL) suppressed GPRC5A expression, whereas deletion mutants, including p65-(1–440) and p65-(296–551), did not (Figure 3D). These suggest that both RHD and TAD of p65 are required for GPRC5A repression.

Posttranslational modifications, including phosphorylation, are known to play an important role in regulating the transactivation activities of RelA/p65 (18–20). Previously, it was shown that phosphorylation of serine 276 is critical for p65-mediated repression of breast cancer metastasis suppressor 1 (BRMS1) (21). We asked if serine 276 is essential for repression of GPRC5A. To determine the role of serine 276 of p65, we established inducible clones, expressing either wild-type (WT) p65 or p65-S276A mutant in Calu-1 cells. Immunoblot analysis showed that WT-p65 suppressed GPRC5A expression whereas p65-S276A did not (Figure 3E). Consistently, qPCR analysis showed that expression of WT-p65 repressed GPRC5A at the mRNA level, whereas p65-S276A mutant did not (Figure 3F). Taken together, the serine 276 site in the RHD of p65 is critical for NF-κB–mediated repression of GPRC5A.

### NF-κB–mediated GPRC5A repression is independent of its transactivation functions.

**Figure 4:**
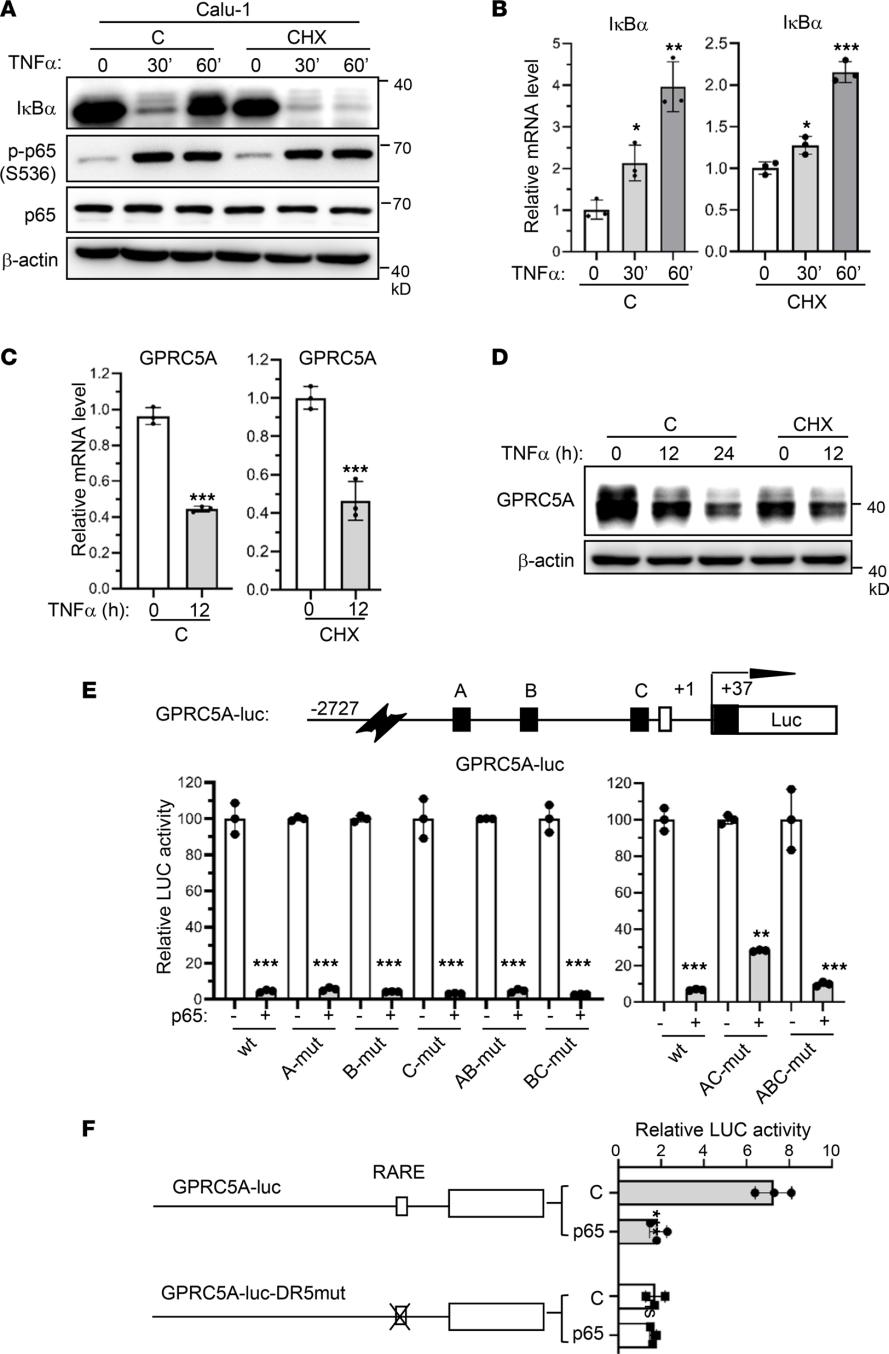
NF-κB–mediated repression of GPRC5A is independent of transcription activation. (**A** and **B**) Calu-1 cells were treated with TNF-α and CHX separately or in combination; IκBα protein and mRNA levels were determined by Western blotting and quantified with ImageJ (NIH) (**A**) and qPCR (**B**), respectively. Data are presented as the mean ± SD. (**C** and **D**) Calu-1 cells were treated with TNF-α and CHX separately or in combination; GPRC5A mRNA and protein levels were determined by qPCR (**C**) and Western blotting and quantified with ImageJ (**D**), respectively. Data are presented as the mean ± SD. (**E**) Three NF-κB binding sites (designated as letters A–C) on GPRC5A promoter–luc plasmid were mutated individually or in combination. The repression effect of p65 was determined by luciferase assay. (**F**) The RA response element (RARE) at the GPRC5A promoter–luc plasmid was mutated, and the repression effect of p65 was determined by luciferase assay. Data are presented as mean ± SD from 3 independent experiments with duplicates and analyzed by 2-tailed Student’s *t* test, *P < 0.05; **P < 0.01; ***P < 0.001.

NF-κB is known to act as a transcription activator. One possibility is that NF-κB–mediated repression is through transactivation of its downstream target genes, which indirectly repress GPRC5A expression. To test this possibility, we examined the effects of protein translation inhibitor cycloheximide (CHX) on NF-κB–induced transactivation and transrepression. Immunoblot analysis showed that TNF-α induced IκBα degradation at 30 minutes in Calu-1 cells (Figure 4A), via an ubiquitination pathway (22, 23). The IκBα level was restored at 60 minutes through de novo protein synthesis (Figure 4A) since IκBα is a well-known NF-κB target gene (22). Although CHX treatment blocked IκBα expression at 60 minutes (Figure 4A), it did not block the IκBα mRNA level (Figure 4B), indicating that CHX treatment indeed blocked new protein synthesis. Importantly, CHX treatment, which blocks protein synthesis, did not alter TNF-α–induced GPRC5A repression at both the mRNA (Figure 4C) and protein levels (Figure 4D). These results suggest that transactivation of NF-κB downstream target genes is not required for TNF-α–induced GPRC5A repression. In addition, treatment with proteasome inhibitor MG132 and autophagy inhibitor chloroquine had no effect on GPRC5A level following TNF-α treatment or p65 induction (Supplemental Figure 1; supplemental material available online with this article; https://doi.org/10.1172/jci.insight.153976DS1), supporting that NF-κB–mediated GPRC5A repression is regulated at the transcriptional level. Taken together, NF-κB represses GPRC5A expression at the transcriptional level, which is independent of transactivation of NF-κB downstream target genes.

Next, we asked whether NF-κB acts via direct binding to the potential NF-κB response cis-element (NFRE) in the GPRC5A promoter. By screening potential NFRE-like sequences in the GPRC5A promoter using software, we found 3 potential NFRE sites in the promoter region. To determine whether these sites (designated as A, B, and C; Figure 4E, upper) play a role in NF-κB–mediated transcriptional GPRC5A repression, we examined the effect of p65 on GPRC5A promoter–driven luciferase (GPRC5A-luc) reporters with various mutations on these sites. Transfection of HEK293T cells with various constructs showed that p65 repressed the luciferase activity from GPRC5A-luc. Importantly, mutation of any of the 3 potential sites or different combinations of mutations did not affect p65-mediated repression of GPRC5A-luc reporters (Figure 4E, lower). This suggests that none of the 3 potential NF-κB–binding cis-elements is required for NF-κB–mediated repression. Instead, we found that the construct GPRC5A-luc-DR5mut, which has a mutated retinoic acid response element (RARE) in the promoter of GPRC5A (17), completely lost luciferase activity regardless of the expression of the NF-κB p65 subunit (Figure 4F). This suggests that RARE at the GPRC5A promoter is critical for regulating its expression and raises an interesting question of whether NF-κB–mediated repression of GPRC5A is by disrupting the transcription complex at RARE in its promoter.

### NF-κB represses GPRC5A via inhibiting RA signaling.

**Figure 5:**
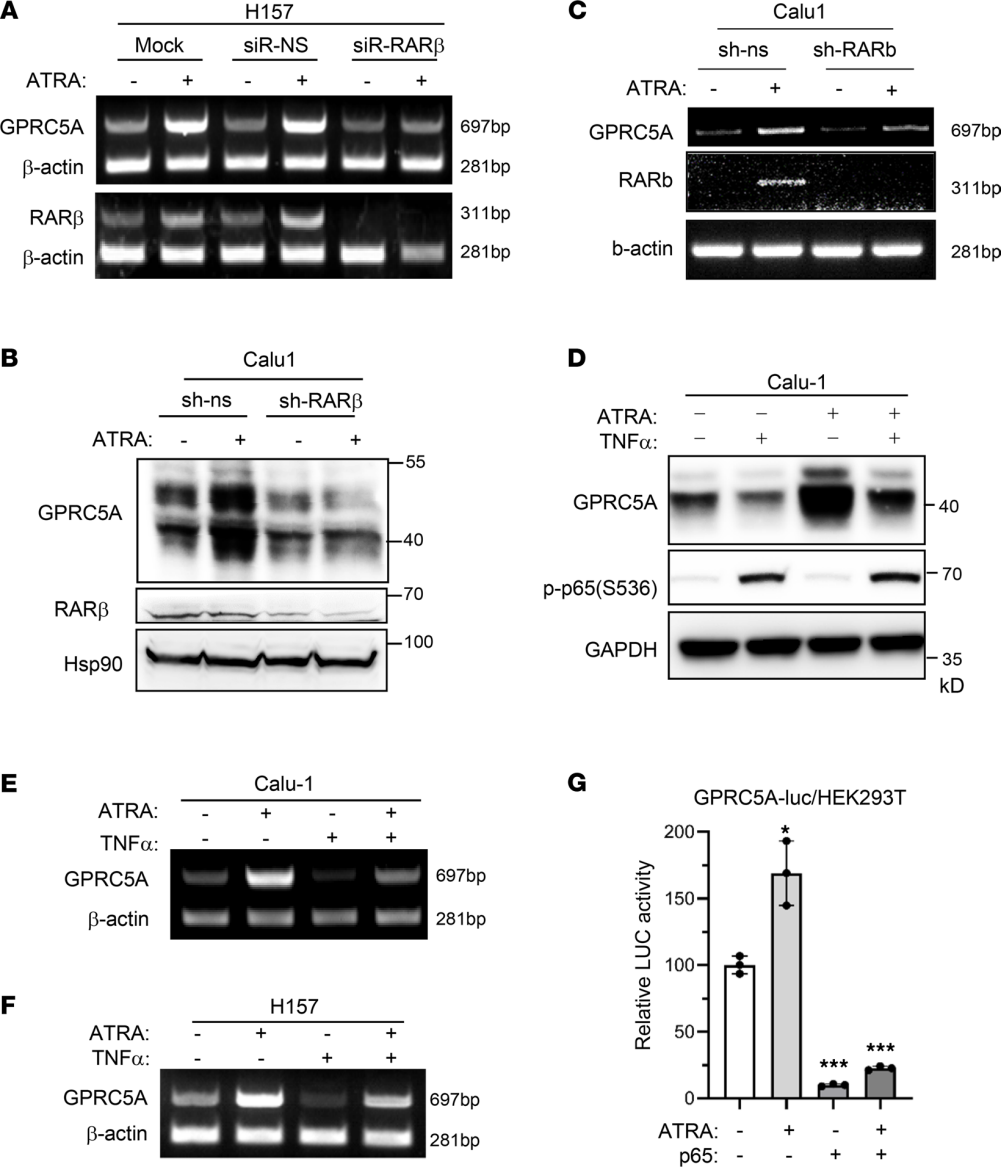
NF-κB inhibits RA-induced GPRC5A expression. (**A**) RARβ in H157 cells was knocked down by siRNA followed by treatment with or without ATRA. The mRNA levels of RARβ and GPRC5A were determined by RT-PCR and quantified by ImageJ. (**B** and **C**) RARβ in H157 cells was knocked down by RARβ short hairpin (sh) RNA followed by treatment with or without ATRA. Protein and mRNA levels of RARβ and GPRC5A were determined by Western blotting (**B**) and RT-PCR and quantified by ImageJ (**C**), respectively. (**D** and **E**) Calu-1 cells were treated with TNF-α and ATRA separately or in combination, and the GPRC5A protein and mRNA levels were determined by Western blotting (**D**) and RT-PCR and quantified by ImageJ (**E**). (**F**) H157 cells was treated with TNF-α and ATRA separately or in combination. GPRC5A mRNA levels were determined by RT-PCR and quantified by ImageJ. (**G**) HEK293T cells were transfected with GPRC5A-luc and p65 plasmids and treated with or without ATRA. The p65 repression effect was determined by luciferase assay. Data are presented as mean ± SD from 3 independent experiments with duplicates and analyzed by 2-tailed Student’s *t* test, *P < 0.05; ***P < 0.001.

Since GPRC5A is an RA target gene, we asked whether NF-κB represses GPRC5A expression via inhibiting RA-mediated transcription. First, we examined the role of the RA signaling pathway in GPRC5A expression. ATRA treatment induced both GPRC5A and RARβ in NSCLC cell line H157 by reverse transcription PCR (RT-PCR) analysis; however, the induction was eliminated when RARβ was knocked down by siRNA (Figure 5A). These results suggest that RARβ is essential for GPRC5A expression. Consistently, immunoblot analysis showed that ATRA could induce GPRC5A in H157-vector cells, but this induction was eliminated in H157-antisense (-ASβ) cells, in which RARβ was blocked by RARβ ASβ RNA (24) (Figure 5B). The inhibition was at the mRNA level since induction of GPRC5A mRNA was eliminated in H157-ASβ cells (Figure 5C). These results suggest that RARβ and RA signaling are essential for GPRC5A expression.

Next, we examined the effect of TNF-α on ATRA-induced GPRC5A expression. Immunoblot showed that ATRA treatment induced GPRC5A expression in Calu-1 cells (Figure 5D). Importantly, TNF-α treatment substantially inhibited GPRC5A expression at both basal and RA-induced levels (Figure 5D). Consistently, RT-PCR analysis validated that TNF-α–mediated GPRC5A repression was at the mRNA level (Figure 5E). Similar effects were observed in H157 cells, in which GPRC5A at both basal and ATRA-induced levels was substantially repressed by TNF-α (Figure 5F). Consistently, cotransfection of p65 suppressed both basal and ATRA-induced luciferase activities in GPRC5A-luc reporter in HEK293T cells (Figure 5G). Taken together, RA-induced GPRC5A is repressed by NF-κB signaling.

### RelA/p65 interacts with RARα/β and is recruited to the RARE at the GPRC5A promoter.

**Figure 6:**
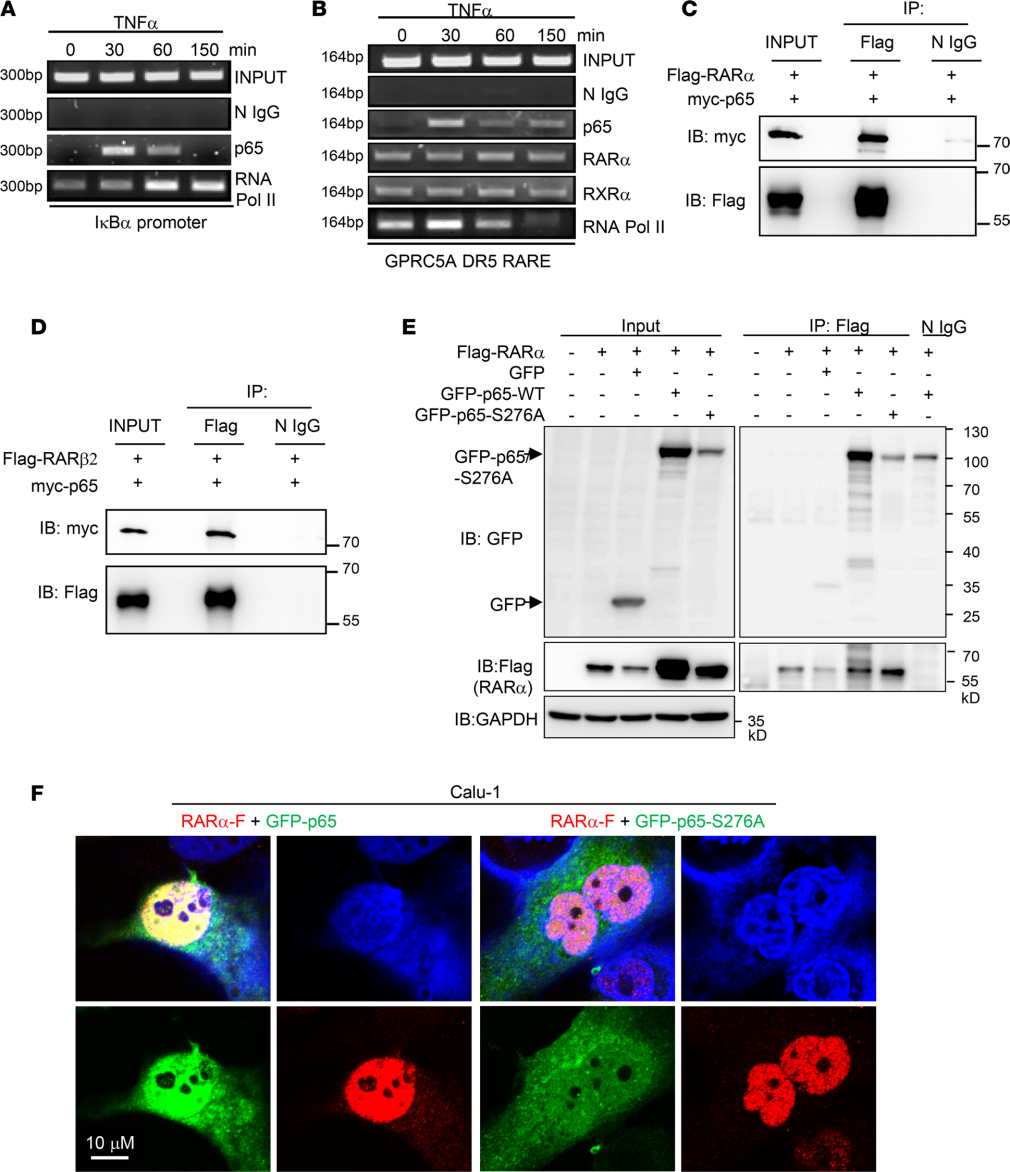
NF-κB subunit p65 is recruited to the promoter of GPRC5A via physically interacting with RAR. (**A** and **B**) Calu-1 cells were treated with TNF-α (10 ng/mL) for various time points. The change of p65 and other proteins as indicated in the figure binding to the IκBα promoter (**A**) and GPRC5A promoter (**B**) was analyzed by chromatin immunoprecipitation (ChIP) assay using corresponding specific antibodies. Input as positive control and normal IgG (N IgG) as negative control. (**C** and **D**) The interaction of p65 with RARα (**C**) and RARβ2/β4 (**D**) was determined by immunoprecipitation (IP) assay. (**E**) RARα-Flag–expressing plasmid was cotransfected with GFP/GFP-p65/GFP-p65-S276A mutant to HEK293T cells. After 48 hours, cells were lysed by RIPA, and IP assay was performed to detect the interaction between RARα and WT RelA/p65 or serine 276A mutant. (**F**) Representative images of IF analysis of Calu-1 cells transfected with RARα-Flag plus GFP-p65 or GFP-p65-S276A as indicated. Scale bar: 10 μm.

GPRC5A is a target gene of RA (5, 16, 17); ATRA treatment induces the association of RARα/β to RARE at the GPRC5A promoter (17). To determine the mechanism of NF-κB–mediated GPRC5A repression, we examined the binding of NF-κB at the GPRC5A promoter following TNF-α treatment via chromatin immunoprecipitation (ChIP) assay. For comparison, we first examined the recruitment of p65 to the IκBα promoter. ChIP analysis showed that p65 was recruited to the IκBα promoter after 30 minutes of TNF-α treatment, which was followed by enhanced recruitment of RNA polymerase II at 60 and 150 minutes (Figure 6A), indicating that NF-κB induces the assembly of transcription machinery at the IκBα promoter. Next, we examined the effect of NF-κB association at the GPRC5A promoter. Although TNF-α treatment induced p65 recruitment to the RARE, it substantially suppressed the recruitment of RNA polymerase II to the RARE at the GPRC5A promoter at the 150-minute interval compared with 0 and 30 minutes (Figure 6B). In comparison, the binding of RARα and retinoic X receptor α (RXRα) to the GPRC5A promoter was not changed (Figure 6B). This suggests that TNF-α treatment disrupts the assembly of RNA polymerase II complex after p65 is recruited to the RARE at the GPRC5A promoter.

Since the cis-element RARE is recognized by the trans-element RAR/RXR complex, it raises a question of whether recruitment of NF-κB to RARE is through the RAR/RXR complex. Next, we examined the interaction between RARα and p65 via immunoprecipitation (IP) assay. IP immunoblot analysis showed that IP Flag-tagged RARα pulled down myc-tagged p65 (Figure 6C). This suggests that p65 can interact with RARα. Similarly, IP Flag-tagged RARβ2 and RARβ4 also pulled down myc-tagged p65 (Figure 6D), indicating that p65 can also interact with RARβ. Thus, NF-κB can interact with the RARα/β complex. Because p65-S276A lost the ability to repress GPRC5A, we then asked if p65-S276A is able to interact with the RAR complex. IP immunoblot analysis showed that although IP Flag-RARα pulled down GFP-p65, it failed to pull down GFP-p65-S276A (Figure 6E). Consistently, immunofluorescence (IF) staining analysis showed that GFP-p65 (shown in green) was highly colocalized with RARα-Flag (shown in red) in transfected Calu-1 cells, whereas GFP-p65-S276A (green) was mainly located in cytoplasm, not colocalized with RARα-Flag (red) (Figure 6F). These data suggest that the serine 276 of p65 is essential for interaction with RARα. Taken together, these results suggest that p65, through interaction with RARα/β, is recruited to RARE at the GPRC5A promoter, leading to disrupted assembly of RNA polymerase II, resulting in suppressed transcription.

### Activated NF-κB inhibits acetylated histone H3K9 in GPRC5A promoter.

**Figure 7:**
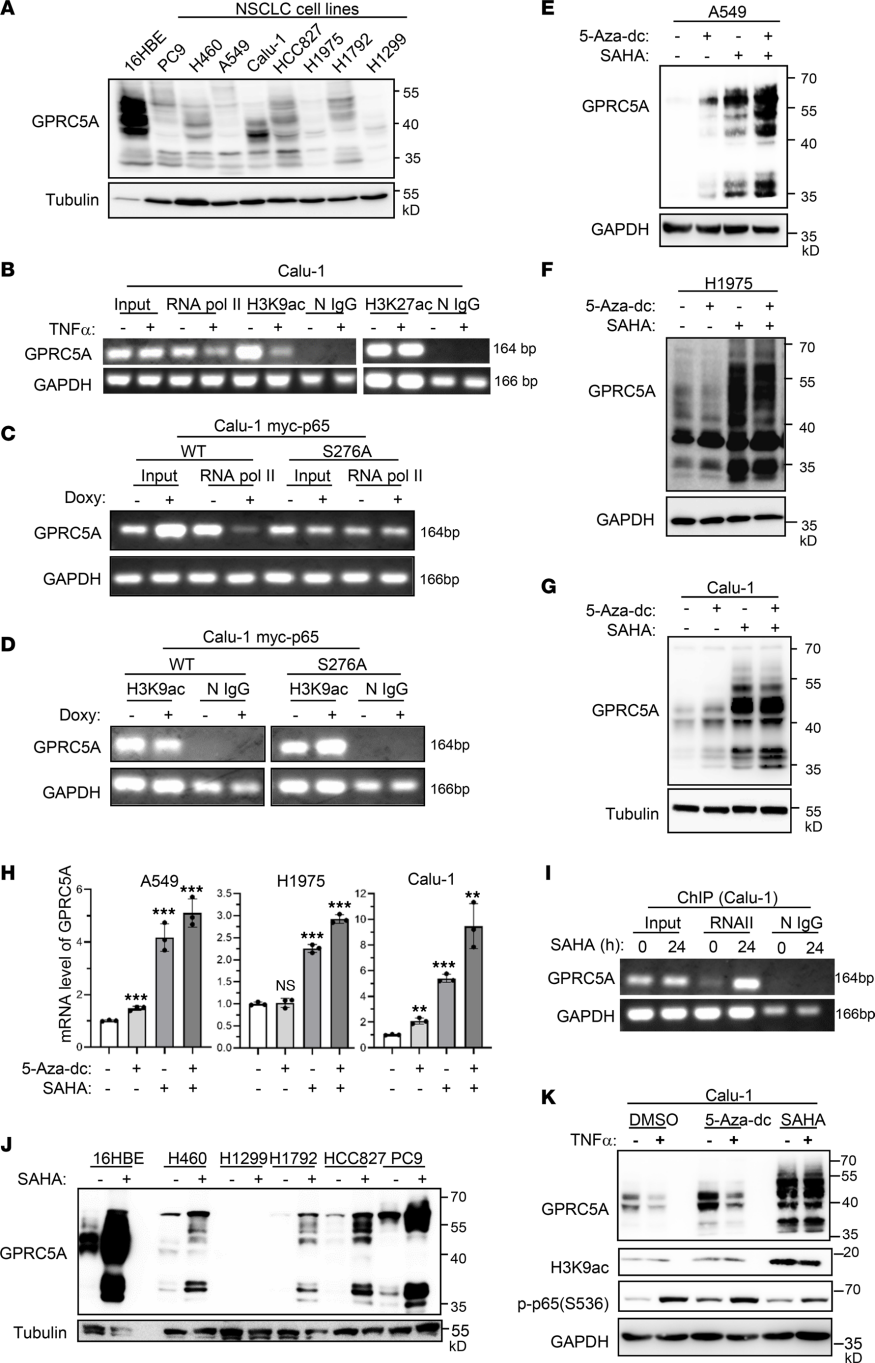
NF-κB–mediated GPRC5A suppression is associated with epigenetic alteration. (**A**) GPRC5A protein expression level in multiple human NSCLC cell lines and normal human bronchial epithelial cell line (16HBE) was analyzed by Western blotting. (**B**) Calu-1 cells treated with or without TNF-α (10 ng/mL) for 12 hours. Binding of RNA polymerase II and the histone modification at the GPRC5A promoter were analyzed by ChIP using specific antibodies. (**C** and **D**) Calu-1 cells with inducible expression of WT and serine 276A mutant p65 were treated with doxycycline (300 ng/mL) for 12 hours; the change of RNA polymerase II binding (**C**) and histone modification (H3K9ac) at the GPRC5A promoter (**D**) were analyzed by ChIP. Input as positive control and normal IgG (N IgG) as negative control. (**E**–**H**) A549, H1975, and Calu-1 cells were treated with 5-Aza-dc (1 μM, 4 days) or SAHA (2.5 μM, 24 hours) individually or in combination; GPRC5A protein (**E**–**G**) and mRNA levels (**H**) were analyzed via Western blotting and qPCR. Data are presented as the mean ± SD. (**I**) Calu-1 cells were treated with or without SAHA (2.5 μM) for 12 hours; RNA polymerase II binding at the GPRC5A promoter was analyzed by ChIP. (**J**) Normal human bronchial epithelial cell line (16HBE) and multiple human NSCLC cell lines were treated with or without SAHA (2.5 μM, 24 hours); GPRC5A protein levels were analyzed by Western blotting. (**K**) Calu-1 cells were pretreated with DMSO (as vehicle control), 5-Aza-dc (1 μM, 3 days) or SAHA (2.5 μM, 3 hours) followed by TNF-α (10 ng/mL) treatment for an additional 24 hours. GPRC5A protein levels were analyzed by Western blotting. All data are presented as mean ± SD from 3 independent experiments with duplicates and analyzed by 2-tailed Student’s *t* test, **P < 0.01; ***P < 0.001. H3K9ac, acetylated histone H3K9.

To determine the general mechanism of GPRC5A repression in NSCLCs, we examined GPRC5A’s expression in 8 NSCLC cell lines and a normal lung epithelial cell line, 16HBE, via immunoblot analysis. The results showed that GPRC5A was repressed in most of the NSCLC cell lines under normal culture conditions (Figure 7A), suggesting that epigenetic repression of GPRC5A is prevalent in NSCLCs (6, 25).

DNA methylation and histone posttranslation modification are the major mechanisms involved in epigenetic silence of tumor suppressor genes (21, 26). To determine the potential role of DNA methylation in GPRC5A repression, we examined the status of DNA methylation in 2 CpG islands at the GPRC5A promoter in lung cancer tissues and AN tissues via bisulfite sequencing PCR. We found that there was no significant difference in GPRC5A promoter methylation between lung cancer and AN tissues (Supplemental Figure 2). Moreover, we found that overexpression of p65 did not significantly alter the DNA methylation status in Calu-1 cells (Supplemental Figure 3). These results suggest that DNA methylation is unlikely to play an important role in GPRC5A repression in NSCLCs.

Next, we asked if posttranslation modification of histones plays a role in GPRC5A repression and whether it is induced by NF-κB. ChIP assay showed that TNF-α treatment substantially suppressed RNA polymerase II assembly in the GPRC5A promoter (Figure 7B); noticeably, H3K9ac, an active marker of gene expression, was also substantially suppressed in the GPRC5A promoter following TNF-α treatment. In comparison, H3K27ac at the GPRC5A promoter was not changed following TNF-α treatment (Figure 7B). To extend the analysis further, we also examined the effects of NF-κB mutants in histone modification. Overexpression of WT-p65, but not S276A-p65, substantially inhibited the recruitment of RNA polymerase II at the GPRC5A promoter in Calu-1 cells (Figure 7C); importantly, expression of WT-p65 inhibited H3K9ac, whereas expression of p65-S276A did not (Figure 7D). These results suggest that NF-κB inhibits both RNA polymerase II assembly and H3K9ac at the GPRC5A promoter. Thus, NF-κB–induced suppression of H3K9ac is involved in epigenetic repression of GPRC5A.

### Histone deacetylation but not DNA methylation is mainly responsible for epigenetic repression of GPRC5A in NSCLCs.

To determine the full extent of the mechanism underpinning the epigenetic repression of GPRC5A, we examined the roles of DNA (cytosine-5)-methyltransferase 1 (DNMT1) inhibitor 5-Aza-2-dc and pan-histone deacetylase (pan-HDAC) inhibitor SAHA in NSCLC cell lines. The results showed that SAHA treatment drastically restored GPRC5A expression in A549, H1975, and Calu-1, whereas 5-Aza-dc had limited effect (Figure 7, E–G). qPCR analysis indicated that restoration of GPRC5A expression via various treatments was at the mRNA level (Figure 7H). Treatment with 5-Aza-2-dc had limited effect on GPRC5A mRNA and RARβ mRNA in these NSCLC cell lines (Supplemental Figure 4, A and B). These findings further support that NF-κB–induced histone modification, rather than DNA methylation, plays a major role in epigenetic repression of GPRC5A. ChIP assay showed that SAHA treatment substantially enhanced the recruitment of RNA polymerase II to the GPRC5A promoter in Calu-1 cells (Figure 7I), supporting the key role HDACs play in GPRC5A repression. Consistently, SAHA-mediated restoration of GPRC5A was also observed in other NSCLC cell lines, including H460, H1792, HCC827, and PC9, and normal cell line 16HBE, though not in H1299 (Figure 7J and Supplemental Figure 5). In addition, TNF-α treatment suppressed GPRC5A expression in the presence of 5-Aza-dc, but not in the presence of SAHA (Figure 7K). These findings suggest that SAHA abrogates TNF-α–mediated repression of GPRC5A, whereas 5-Aza-dc does not. Taken together, NF-κB–mediated histone deacetylation, rather than DNA methylation, is mainly responsible for epigenetic repression of GPRC5A. To determine the biological effects of the histone deacetylation inhibitor in vivo, A549 cells were s.c. inoculated in nude mice, then treated with SAHA or placebo. SAHA treatment significantly suppressed tumor growth (Supplemental Figure 6, A–C) and restored GPRC5A expression compared with the placebo group (Supplemental Figure 6, D and E). These results strongly support the notion that histone deacetylation plays a major role in the epigenetic repression of GPRC5A.

To determine the biological roles of GPRC5A deficiency on lung epithelial cells, we first enhanced the transformed phenotype of mouse tracheal epithelial cells (MTECs) by repeated treatment with tobacco carcinogen NNK (100 pM for 10 passages), then performed biological characterization of MTEC-NNK10 cells derived from this treatment. The results showed that MTEC-KO-NNK10 cells formed more and bigger colonies in soft agar than MTEC-WT-NNK10 cells did (Supplemental Figure 7, A and B). This suggests that GPRC5A deficiency enhances the transforming phenotype of lung epithelial cells. Taken together, NF-κB induces GPRC5A repression via histone deacetylation, whereas GPRC5A repression enhances oncogenic features in lung epithelial cells.

## Discussion

In this study, we showed that NF-κB can function as a transcriptional repressor on GPRC5A expression via a transactivation-independent mechanism. NF-κB–mediated GPRC5A repression is through interaction with RARα/β, resulting in the suppression of RNA polymerase II recruitment in RARE at the GPRC5A promoter. Moreover, NF-κB inhibits histone H3K9ac, rather than induces DNA hypermethylation, at the GPRC5A promoter. Consistently, SAHA treatment largely restores GPRC5A expression in NSCLC cells, whereas treatment with 5-Aza-dc has little effect. Because Gprc5a deficiency enhances the transformed phenotype of lung epithelial cells, NF-κB–mediated GPRC5A repression contributes to neoplasia of lung epithelial cells.

Chronic inflammation is associated with neoplasia and COPD. Generally, individuals with chronic pulmonary inflammation have an increased incidence of lung cancer development. In fact, patients with COPD have about 5 times greater incidence of lung cancer development than healthy individuals. Bacterial colonization, particularly nontypeable *Haemophilus influenzae* (NTHi) (Gram-negative, G^–^, bacteria), is implicated as a cause of airway inflammation in COPD. LPS endotoxin of G^–^ bacteria is a potent inflammatory inducer. Extrinsic lung inflammation induced by repeated administration of NTHi, in combination with tobacco carcinogen NNK, enhances lung tumorigenesis in *Gprc5a*-KO mice (27). These observations strongly suggest that chronic inflammation promotes lung tumorigenesis.

NF-κB is a well-known transcriptional activator (28). However, little is known about its role as a transcriptional repressor. Previously, it was reported that RelA/p65 can act as a transcriptional repressor to repress metastasis suppressor gene BRMS1. Mechanistically, NF-κB binds the specific κB binding site at the BRMS1 promoter and recruits DNMT1, which mediates DNA methylation in CpG islands located at the promoter region, resulting in epigenetic repression (21). Nevertheless, the mechanism of NF-κB–mediated BRMS1 repression is via induction of DNA methylation, which is distinct from the repression of GPRC5A via histone deacetylation found in this study.

RA signaling plays an important role in the regulation of cell proliferation, differentiation, and homeostasis. RA exerts its role by interacting with nuclear RAR and RXRs. GPRC5A is an RA target. Thus, the status of RARs is crucial for GPRC5A expression. Of note, RARβ expression is often lost or reduced in a large percentage of lung cancer (29, 30). For example, methylation of the RARβ2 promoter is found in 40% of NSCLCs. Thus, this raises an interesting question of whether hypermethylation of RARβ plays an important role in GPRC5A repression. Although 5-Aza-2-dc treatment led to minor increase of RARβ mRNA in NSCLC cells, it had no effect on the level of GPRC5A mRNA, consistent with the observation of no DNA methylation at the GPRC5A promoter. In contrast, SAHA treatment greatly elevated the levels of GPRC5A mRNA, supporting the critical role of H3K9ac in regulating GPRC5A expression. Although the combination of 5-Aza-dc and SAHA further increased GPRC5A mRNA, the additive effect of 5-Aza-dc on GPRC5A expression was likely mediated by the minor increase of RARβ, which indirectly increased GPRC5A expression.

NF-κB has been found to crosstalk with other signaling pathways, including EGFR (31), p53 (32), HIF1α (33), and glucocorticoid signaling (34). It was shown that vitamin A deficiency enhances inflammatory response or NF-κB activation in vivo, whereas administration of ATRA reduces inflammatory response or NF-κB activation in animal models (35). These findings suggest that vitamin A or RA signaling inhibits NF-κB signaling. Moreover, the p50 and p65 components of NF-κB were shown to bind to RXR in a ligand-independent manner, and RXR can inhibit NF-κB activation in a ligand-dependent manner (36). These observations suggest that there is a crosstalk between NF-κB and RA signaling. Retinoids play a fundamental role in development and homeostasis (37), whereas disruption of the RA signaling pathway is implicated in neoplasia or cancers (38, 39). Conversely, activation of RA signaling is used as a strategy for cancer prevention and therapy (40). GPRC5A was originally cloned as RAIG1 (5). An RARE at the GPRC5A promoter is critical for its expression (17). Thus, GPRC5A functions as a key mediator of RA signaling for differentiation or homeostasis in lung tissue. Presumably, the mutually inhibitory effects between NF-κB and RARα/β signaling represent a molecular switch at the crossroads of cell fate determination between differentiation and dedifferentiation.

Previously, we showed that *Gprc5a*-KO leads to increased NF-κB activation in lung epithelium, which is associated with lung tumorigenesis in a mouse model. *Gprc5a*-KO mice are susceptible to pulmonary inflammation and LPS-induced acute lung injury (10, 11). However, the regulatory role of Gprc5a deletion on NF-κB activation in lung epithelial cells appears to be indirect since the effects are stronger in vivo than in vitro. In addition, the effect of GPRC5A knockdown on the NF-κB activation in human lung epithelial cells is not as strong as that in mouse lung epithelial cells. It is likely that multiple mechanisms are involved in the crosstalk between GPRC5A and NF-κB.

**Figure 8:**
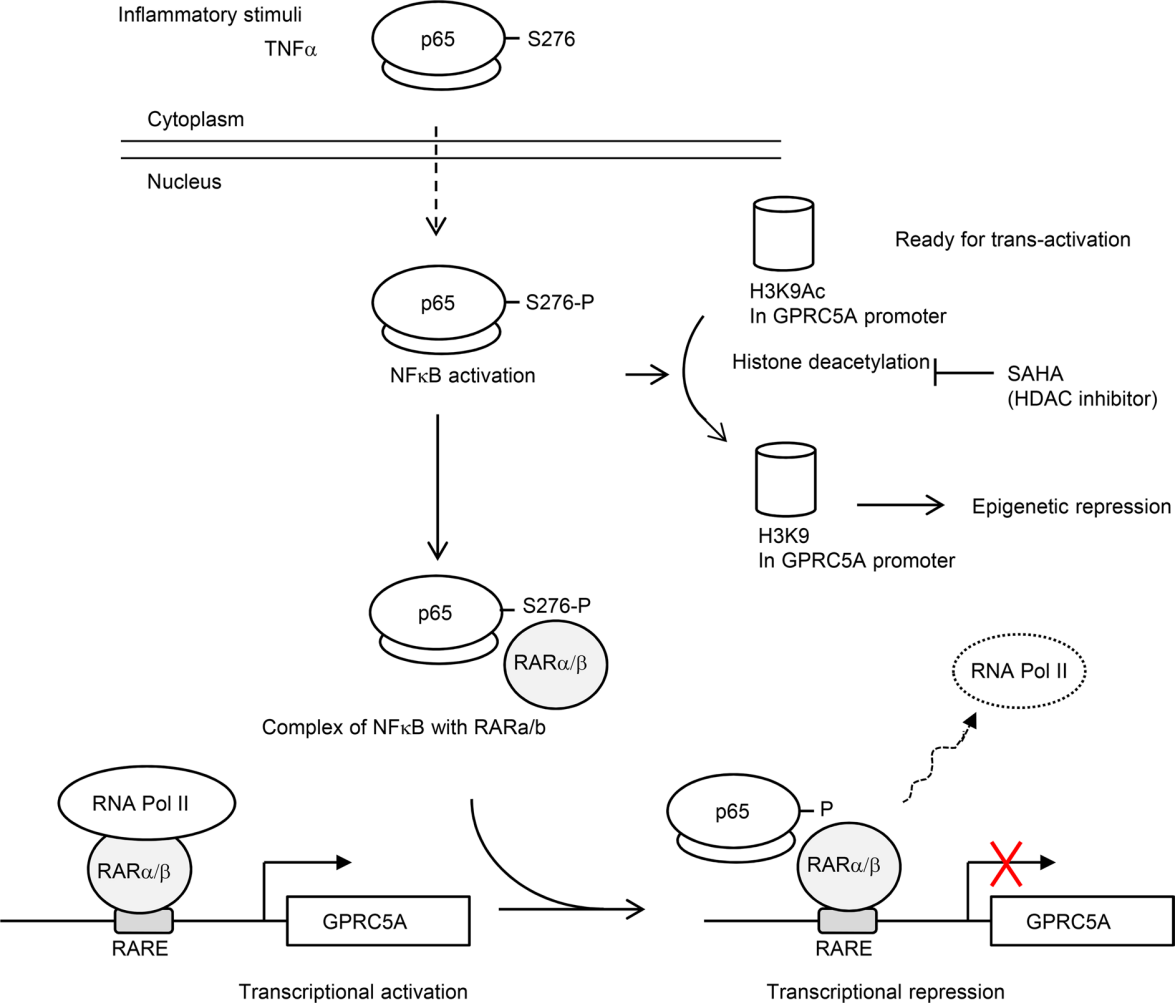
The proposed model of NF-κB–mediated repression of GPRC5A in lung epithelial cells.

Taken together, we find that NF-κB functions as a transcriptional repressor to suppress GPRC5A expression. NF-κB interacts with RARα/β via its transactivation domain, which disrupts the assembly of RAR-mediated RNA polymerase II complex at the GPRC5A promoter, leading to its transcriptional repression. Concurrently, NF-κB induces epigenetic repression via H3K9ac deacetylation at the GPRC5A promoter (Figure 8). Importantly, this epigenetic mechanism of GPRC5A repression is prevalent in NSCLCs. We propose that NF-κB–mediated GPRC5A repression contributes to dedifferentiation or neoplasia in lung epithelial cells.

## Methods

### Cells, reagents, and clinical samples.

Multiple types of NSCLC cell lines were used in this work, including bronchioloalveolar carcinoma (H322), mucoepidermoid pulmonary carcinoma (H292G), epidermoid carcinoma (Calu-1), large cell lung cancer (H460), adenocarcinoma (H157, PC9, HCC827, H1792, H1975), and lung carcinoma (A549, H1299). HEK293T cells and all NSCLC tumor cell lines were obtained from ATCC. The A549 and Calu-1 cells were maintained in DMEM supplemented with 10% fetal bovine serum. All other tumor cell lines were maintained in RPMI-1640 medium (Invitrogen) supplemented with 10% fetal bovine serum. A normal human bronchial epithelial cell line, 16HBE (from the Chinese Academy of Medical Sciences), was maintained in DMEM supplemented with 10% fetal bovine serum. WT and Gprc5a-KO MTECs were obtained from normal tracheal tissue of 3-week-old WT and Gprc5a-KO mice (C57BL/6 129Sv, Shanghai Laboratory Animals Center) as described previously (10, 12). Cells were cultured with keratinocyte-SFM supplemented with EGF (5 ng/mL) and bovine pituitary extract (50 mg/mL; Invitrogen). LPS was purchased from MilliporeSigma; TNF-α was purchased from R&D Systems; doxycycline was purchased from Selleckchem; antibodies (catalog numbers in parentheses) against H3K9ac (ab4441) and myc-tag (ab9232) for ChIP were purchased from Abcam; antibodies against Gprc5a (sc-98885), IκBα (sc-371), GAPDH (sc-365062), RARα (C-20), RARβ (C-19), and GFP (sc-8334) were purchased from Santa Cruz Biotechnology; NF-κB p65 NLS antibody (for IHC, NBP2-24541) was purchased from Novus Biologicals; antibodies against p-p65 (S536; 3033), H3K27Ac (8173), and β-actin conjugated with HRP (12262) were purchased from Cell Signaling Technology; p-p65 (S276) antibody (D155005) was from BBI Life Sciences Corporation; RNA polymerase II antibody (05-623B) was from MilliporeSigma; antibodies against myc-tag (catalog 562-5) and Flag-tag (catalog PM020) were purchased from MBL International Corporation; and customized GPRC5A antibody was generated by Abmart. All human clinical samples were provided by Shanghai Chest Hospital (Shanghai, China) and all informed consents were obtained.

### Animal experiments.

NF-κB–driven luciferase-transgenic mice were administrated with LPS through atomization inhalation (500 μg in 5 mL for each cage per day). d-luciferin was injected i.p. 30 minutes after LPS treatment; another 2 hours later, fluorescence imaging was performed to verify the activation of NF-κB. Mice were sacrificed at day 4, 6, and 8, and lungs were analyzed via Western blot and qPCR assays to examine the protein and mRNA levels of Gprc5a.

### Luciferase reporter assay.

Cells were grown to 50% confluence in a 24-well plate and then cotransfected with reporter gene constructs (GPRC5A-luc or NF-κB–luc) and pcDNA3.1-p65 and its mutants. Plasmid pRL-TK (Promega) was used as the internal control in all transfection assays. All transfections were done with Lipofectamine 2000 (Invitrogen) according to the instructions of the manufacturer (41). Cell extracts were prepared 48 hours after transfection, and luciferase activity was measured using the Dual-Luciferase Reporter Assay System (Promega). All experiments were performed 3 times in triplicate.

### IP assay and Western blot analysis.

Cells or tissues were lysed with RIPA buffer (150 mM NaCl, 1% Nonidet P-40, 0.1% SDS, 0.5% deoxycholate, 50 mM Tris at pH 8.0, 25 mM NaF, 2 mM Na_3_VO_4_, 5 mM PMSF, and 2 mg/mL of aprotinin) (42). IP and Western blot were performed as described previously (43, 44). Briefly, 2 μg Flag antibody was used for 500 μg total whole-cell lysate for each IP. For Western blot, whole-cell lysate containing 30 μg whole protein was mixed with 2× SDS-PAGE–reduced loading buffer and boiled at 95°C for 5 minutes per gel well. Protein samples were separated by SDS-PAGE and transferred onto nitrocellulose membranes for Western blots. Nonspecific binding to antibodies was blocked with 5% nonfat milk at room temperature for 1 hour. Primary antibodies were incubated at 4°C overnight. To remove the residual primary antibody, membranes were washed 3 times in TBS with 0.05% Tween-20 (TBST) while agitating, 5 minutes per wash. Second antibody conjugated with HRP was incubated at room temperature for 1 hour and washed 3 times in TBST while agitating. ECL kit (MilliporeSigma) was used for detection.

### RT-PCR and quantitative real-time PCR.

Total RNA was isolated using the RNAsimple Total RNA Kit (TIANGEN) according to the manufacturer’s instructions. Reverse transcription was performed using FastQuant RT Kit (TIANGEN) according to the manufacturer’s protocol. For duplex RT-PCR, 0.5 μL cDNA from each sample was amplified with the GPRC5A primers (6) plus β-actin competition primers from Ambion in high-fidelity PCR master mix (Roche Applied Science) according to the manufacturer’s instructions. For specific quantitative real-time PCR, experiments were performed using SuperReal PreMix Plus SYBR Green Kit (TIANGEN) according to the manufacturer’s protocol. All primers used for quantitative real-time PCR are listed in Supplemental Table 1.

### IHC staining.

Tissue samples from human lung cancer and COPD and AN tissues were stained with anti-GPRC5A (1:200 dilution) and anti-p65 NLS (1:100 dilution) antibodies, and each sample was scored by an H-score method that combines the values of immune reaction intensity and the percentage of cell staining as described previously (41). Pearson’s correlation analysis was used to analyze the relationship between GPRC5A expression and activated p65; statistical significance was defined as *P* < 0.05.

### ChIP assay.

ChIP assay was performed by using ChIP kits (9003, Cell Signaling Technology) according to the manufacturer’s instructions. The primers used for ChIP are listed in Supplemental Table 2.

### IF.

GFP-p65 or GFP-p65-S276A mutant was cotransfected with RARα-Flag to Calu-1 cells. RARα-Flag was stained with Flag antibody (MilliporeSigma, M2). The procedure of IF was as described previously (43).

### Statistics.

Differences between experimental groups were analyzed by paired 2-tailed Student’s *t* test. All results are presented as mean ± SD, and represented histograms or images were selected based on the average values. *P* < 0.05 was considered significant.

### Study approval.

All human clinical samples were provided by Shanghai Chest Hospital, Shanghai Jiao Tong University School of Medicine. Patients’ written informed consent was obtained, and studies were approved by the Ethics Committee for Medical Research (IRB) of Shanghai Jiao Tong University School of Medicine. All animal experiments were approved by the Institutional Animal Care and Use Committee (IACUC) of Shanghai Jiao Tong University School of Medicine and performed consistently with the IACUC’s rules.

## Author contributions

HS, XY, YL, and S Zhang designed, performed, and analyzed experiments and cowrote the article. DX, BJ, TW, BS, JX, WG, KL, MH, YK, JL, TZ, YW, FY, S Zhong, J Du, and YEC helped with experiments and provided critical tools and advice. BPZ, QW, and J Deng supervised the study and cowrote the manuscript.

## Supplementary Material

Supplemental data

## Version 1 11/22/2022

In-Press Preview

## Version 2 01/10/2023

Electronic publication
