# NF-κB represses retinoic acid receptor–mediated GPRC5A transactivation in lung epithelial cells to promote neoplasia

**DOI:** 10.1172/jci.insight.186558

**Published:** 2024-10-08

**Authors:** Hongyong Song, Xiaofeng Ye, Yueling Liao, Siwei Zhang, Dongliang Xu, Shuangshuang Zhong, Bo Jing, Tong Wang, Beibei Sun, Jianhua Xu, Wenzheng Guo, Kaimi Li, Min Hu, Yanbin Kuang, Jing Ling, Tuo Zhang, Yadi Wu, Jing Du, Feng Yao, Y. Eugene Chin, Qi Wang, Binhua P. Zhou, Jiong Deng

Original citation *JCI Insight*. 2023;8(1):e153976. https://doi.org/10.1172/jci.insight.153976

Citation for this corrigendum: *JCI Insight*. 2024;9(19):e186558. https://doi.org/10.1172/jci.insight.186558

The authors recently became aware that the GPRC5A-stained adjacent normal sample presented in Figure 1A was previously published (1). The image was inadvertently included during the preparation of Figure 1. The correct figure is shown below. The HTML and PDF files have been updated.

The authors regret the error.

## Figures and Tables

**Figure 1:**
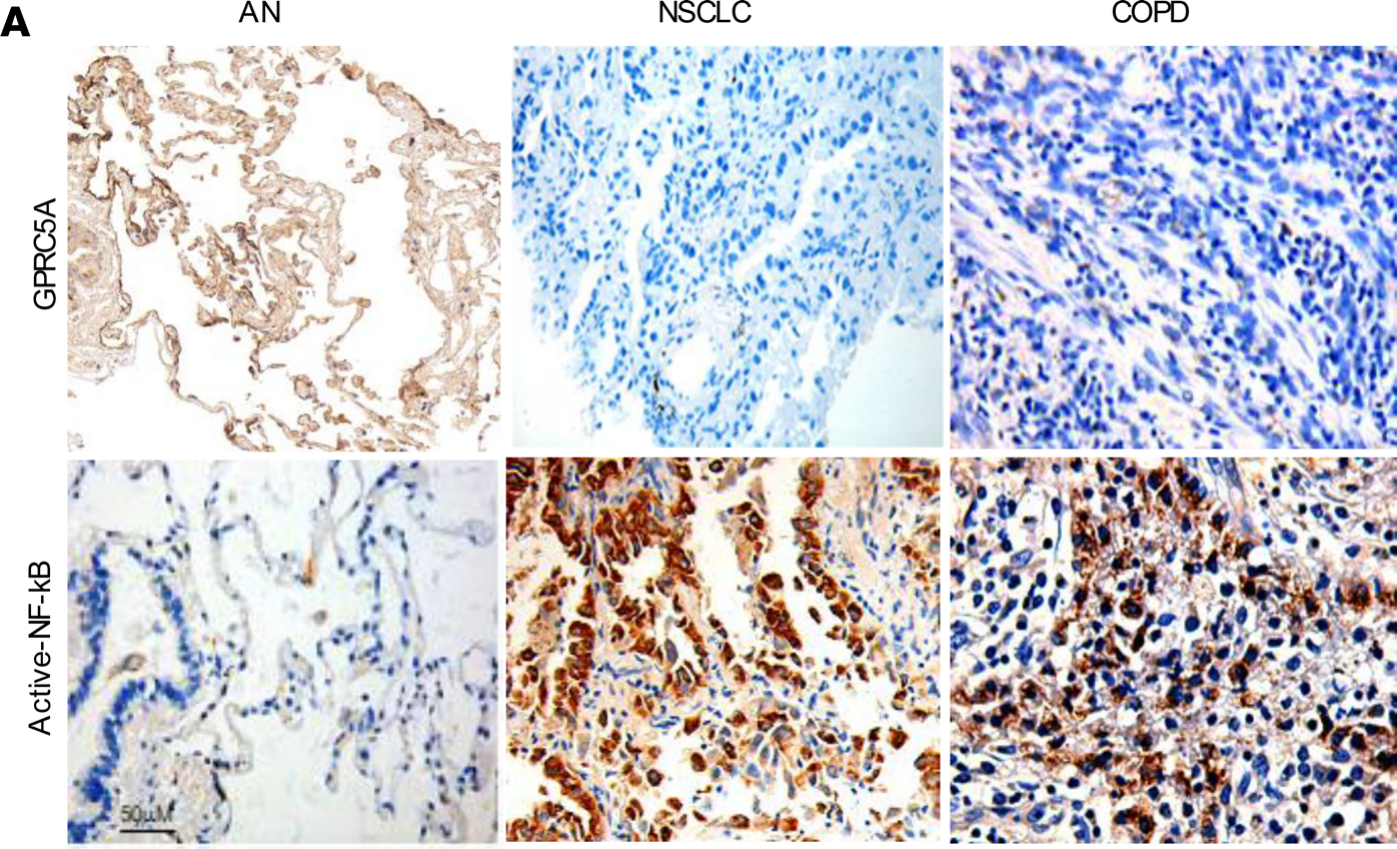


## Version 1 10/08/2024

Electronic publication
