# Supplementary material for: NF-κB represses retinoic acid receptor–mediated GPRC5A transactivation in lung epithelial cells to promote neoplasia
Source: JCI Insight. 2023 Jan 10;8(1):e153976. doi: 10.1172/jci.insight.153976 (PMC9870083; doi:10.1172/jci.insight.153976)
Supplement: Supplemental data [file jciinsight-8-153976-s019.pdf]

Supplementary Information of

NFκB Induces Epigenetic Repression of GPRC5A in Lung Epithelial Cells to Promote Neoplasia

Supplementary Data

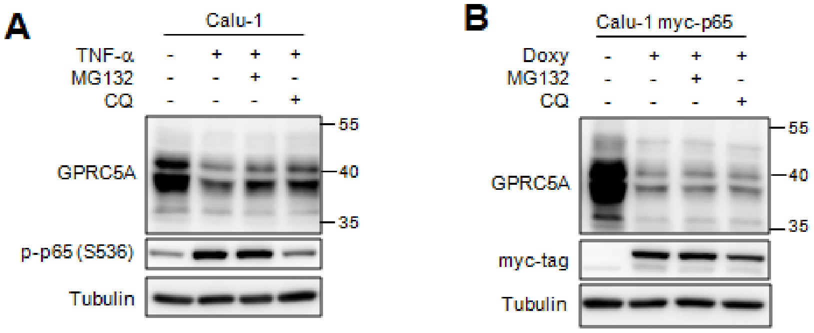

Supplementary Figure 1 Related to Figure 4

Proteasome and autophagy inhibitors do not affect the repression of GPRC5A by TNFα and p65. **A**, Calu-1 cells were treated with TNFα (10 ng/ml) for 3 days and MG132 (10 μM) or chloroquine (CQ) (100 μM) were added to the medium for the last 6 hours, cells were lysed by RIPA. GPRC5A protein levels were analyzed by western blotting. **B**, Calu-1 cells expressed myc-p65 under the control of doxycycline (doxy)-inducible promoter (TRE2) were treated with doxycycline (100 ng/ml) to induce p65 expression for 48h. MG132 (10 μM) or chloroquine (100 μM) were added to the medium for the last 6 hours, cells were lysed by RIPA and GPRC5A protein levels were analyzed by western blotting.

A

```
CpG island 1
AGTGGAGGAAGGCTGGCTTGTCCCGCTGGACTTGACGGCGGAGCTGCTGCAATCTCGCGGGCTGGGCCCTT
GCCCCGGGTGGACCCGAGTGCCTGCGGGGAGGCG GGTGTGGCTCCCCCGGGAGGTGACAGCTGGCGGGGC
CCGTAAGGAGCTGGTCTGCTCCGGCTCCCCTCTCGTAGCCGGCGCCCTGGGACCAGCGCGTGAGCACGTCT
CGGAGGAGTCCGATGCGC TGGGCAGGGCCGGGTGGTCACCCCCGCCCAGCTTCCAGGGG

CpG island 2
GAATAGGCGTGTCCTCTCCCTCGACCCTCCCCCTCCTTGTCCCTCTGCTCACCCCTCGCTCGTTCCCTCCC
TCCGGCGAGGGCCGCCTTTATAACAACTGCTCAGAGTGCGAGGGCGGGATAGCTGTCCAAGGTCTCCCCCA
GCACTGAGGAGCTCGCCTGCTGCCCTCTTGCGCGCGGGAAGCAGCACCAAGTTCACGGCCAACGCCTTGGC
ACTAGGGTAAGTGAGGCGCCAGGAGCCCAGAGCGCTGGGGCCTCCGGATTTAAATCGTCTGGGGAGAGCGG
GGCG TTGGGAGGGGTGGGATAGAAGAGGAGAG
```

B

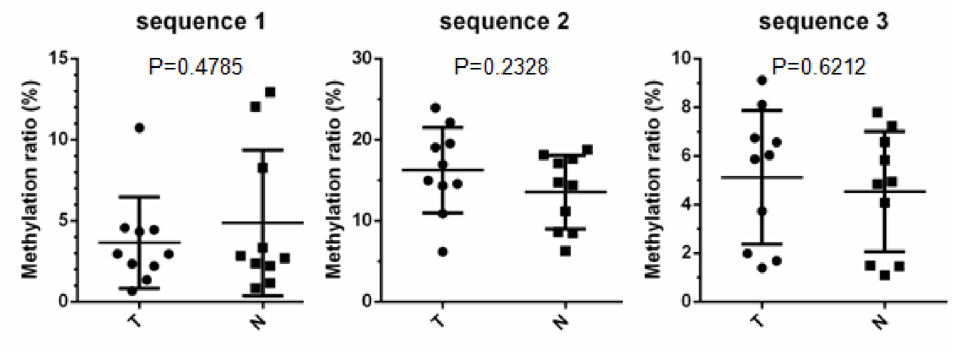

Supplementary Figure 2 Related to Figure 7

DNA methylation at the GPRC5A promoter shows no different between lung cancer and adjacent normal tissues. **A**, three sequences (red color) in 2 CpG islands were chosen to perform DNA methylation sequencing. Sequence 1 and 2 were located in CpG island 1, includes 6 CpG sites respectively. Sequence 3 was located in CpG island 2, includes 5 CpG sites. All three sequences were indicated with red color. **B**, DNA were extract from ten lung cancer and corresponding adjacent normal tissues, DNA methylation levels on these three sequences were analyzed by bisulfite PCR; T, tumor; N, normal.

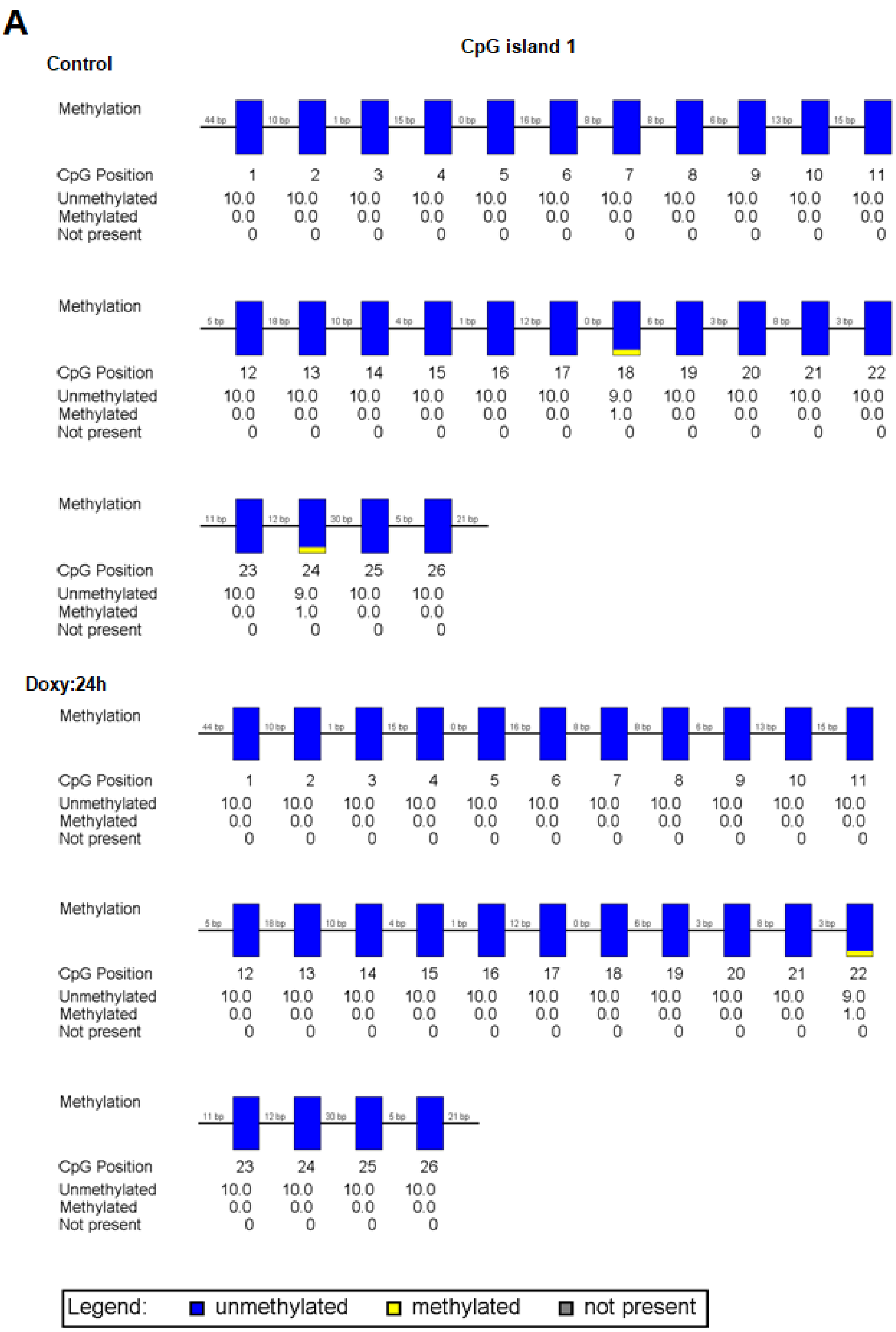
A
CpG island 1
Control
Methylation
CpG Position 1 2 3 4 5 6 7 8 9 10 11
Unmethylated 10.0 10.0 10.0 10.0 10.0 10.0 10.0 10.0 10.0 10.0 10.0
Methylated 0.0 0.0 0.0 0.0 0.0 0.0 0.0 0.0 0.0 0.0 0.0
Not present 0 0 0 0 0 0 0 0 0 0 0
Methylation
CpG Position 12 13 14 15 16 17 18 19 20 21 22
Unmethylated 10.0 10.0 10.0 10.0 10.0 10.0 9.0 10.0 10.0 10.0 10.0
Methylated 0.0 0.0 0.0 0.0 0.0 0.0 1.0 0.0 0.0 0.0 0.0
Not present 0 0 0 0 0 0 0 0 0 0 0
Methylation
CpG Position 23 24 25 26
Unmethylated 10.0 9.0 10.0 10.0
Methylated 0.0 1.0 0.0 0.0
Not present 0 0 0 0
Doxy:24h
Methylation
CpG Position 1 2 3 4 5 6 7 8 9 10 11
Unmethylated 10.0 10.0 10.0 10.0 10.0 10.0 10.0 10.0 10.0 10.0 10.0
Methylated 0.0 0.0 0.0 0.0 0.0 0.0 0.0 0.0 0.0 0.0 0.0
Not present 0 0 0 0 0 0 0 0 0 0 0
Methylation
CpG Position 12 13 14 15 16 17 18 19 20 21 22
Unmethylated 10.0 10.0 10.0 10.0 10.0 10.0 10.0 10.0 10.0 10.0 9.0
Methylated 0.0 0.0 0.0 0.0 0.0 0.0 0.0 0.0 0.0 0.0 1.0
Not present 0 0 0 0 0 0 0 0 0 0 0
Methylation
CpG Position 23 24 25 26
Unmethylated 10.0 10.0 10.0 10.0
Methylated 0.0 0.0 0.0 0.0
Not present 0 0 0 0
Legend: unmethylated methylated not present

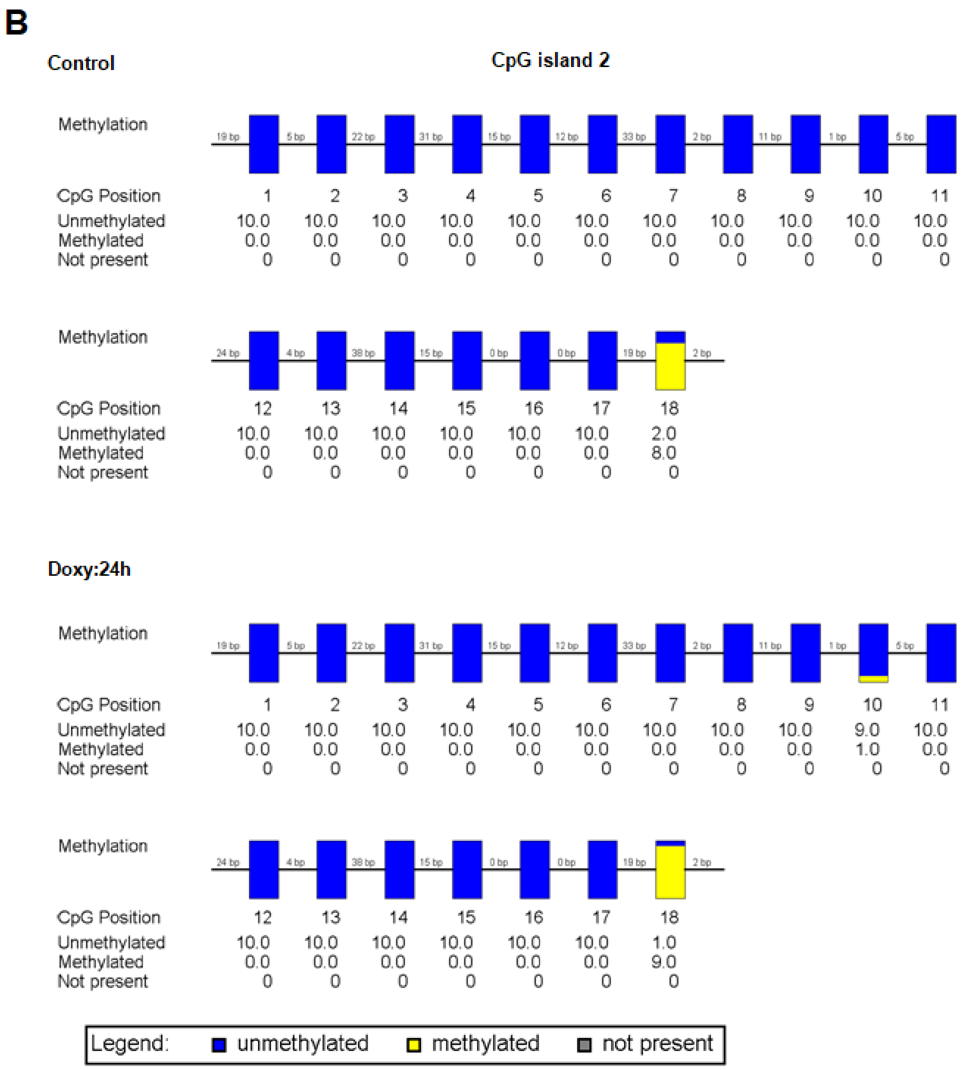

Supplementary Figure 3 Related to Figure 7

Overexpression of p65 in Calu-1 cells do not change the DNA methylation status at the GPRC5A promoter. Calu-1 cells expressed myc-p65 under the control of doxycycline-inducible promoter (TRE2) were treated with DMSO as negative control or doxycycline (300 ng/ml) to induce p65 expression for 24 h. The methylation status of the CpG island 1 (**A**) and CpG island 2 (**B**) at the GPRC5A promoter were analyzed by bisulfite sequencing PCR.

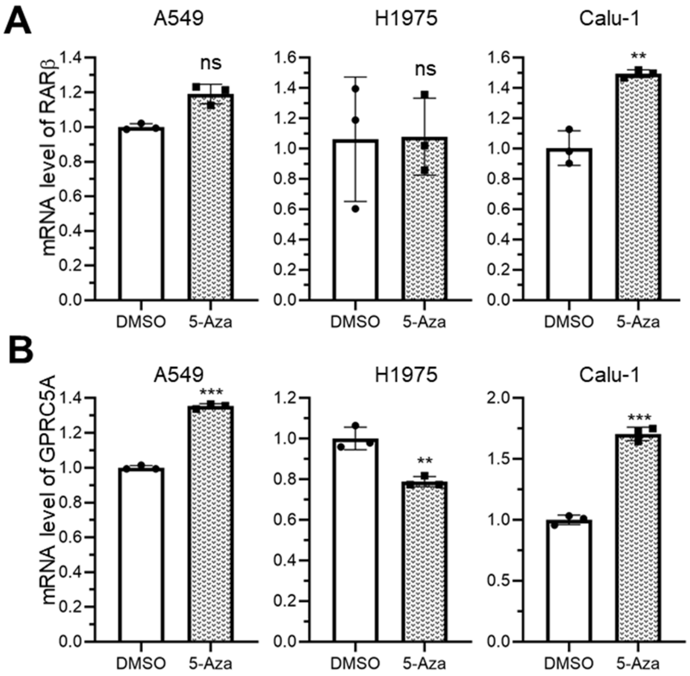

Supplementary Figure 4 Related to Figure 7

5-Aza-dc treatment has limited effects on the levels of RARβ and GPRC5A mRNA in three NSCLC cells. A549, H1975 and Calu-1 cells were treated with or without 5-Aza-dc (1 μM, 4 days). RARβ and GPRC5A mRNA levels were analyzed by quantitative PCR. *p<0.05; **p<0.01; ***p<0.001.

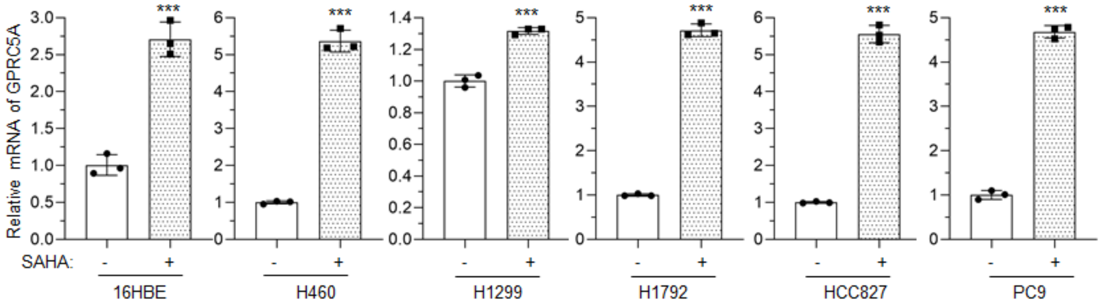

Supplementary Figure 5 Related to Figure 7

SAHA treatment increase GPRC5A mRNA levels. Normal human bronchial epithelial cell line (16HBE) and multiple human NSCLC cell lines were treated with or without SAHA (2.5 μM, 24 hours), GPRC5A mRNA levels were analyzed by quantitative PCR. *p<0.05; **p<0.01; ***p<0.001.

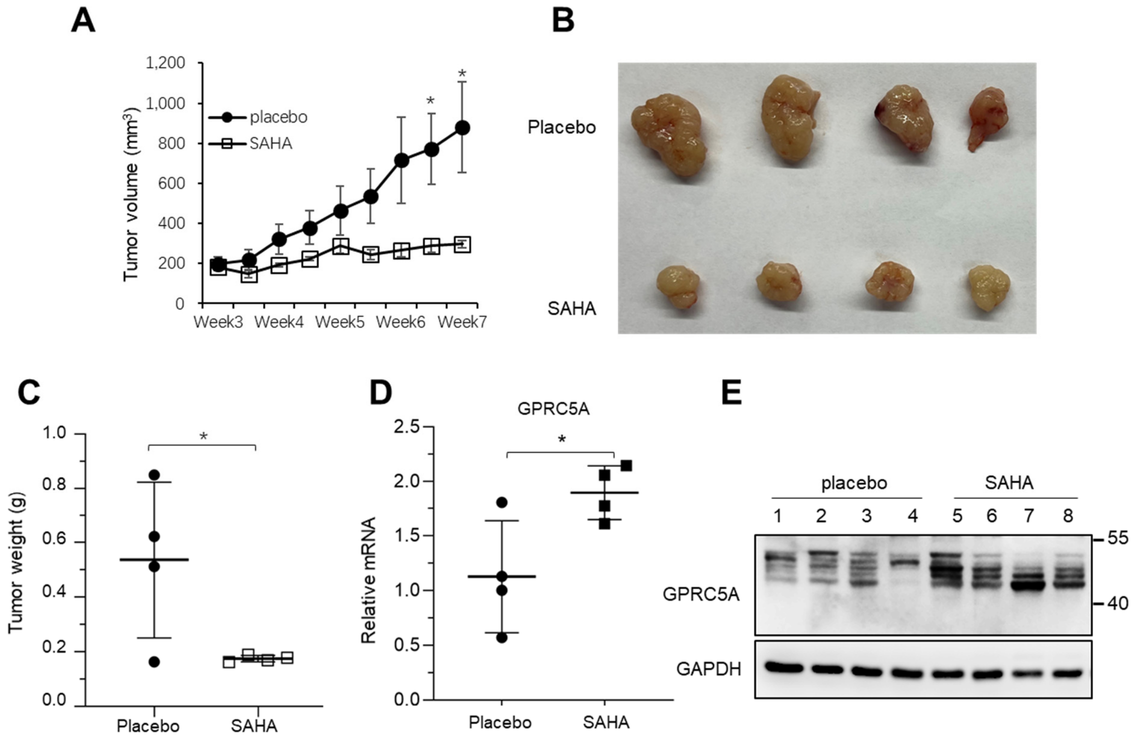

Supplementary Figure 6

SAHA treatment increases GPRC5A expression and inhibits tumor formation in vivo. A549 cells were injected subcutaneously into nude mice; mice were separated to two groups randomly. After three weeks, once group was treated with placebo and the other group was treated with SAHA. Tumor volumes were measured twice a week. After additional four weeks, mice were sacrificed. Tumor weights were measured, and GPRC5A mRNA and protein expression levels were analyzed by qPCR and western blot. *p<0.05; **p<0.01; ***p<0.001.

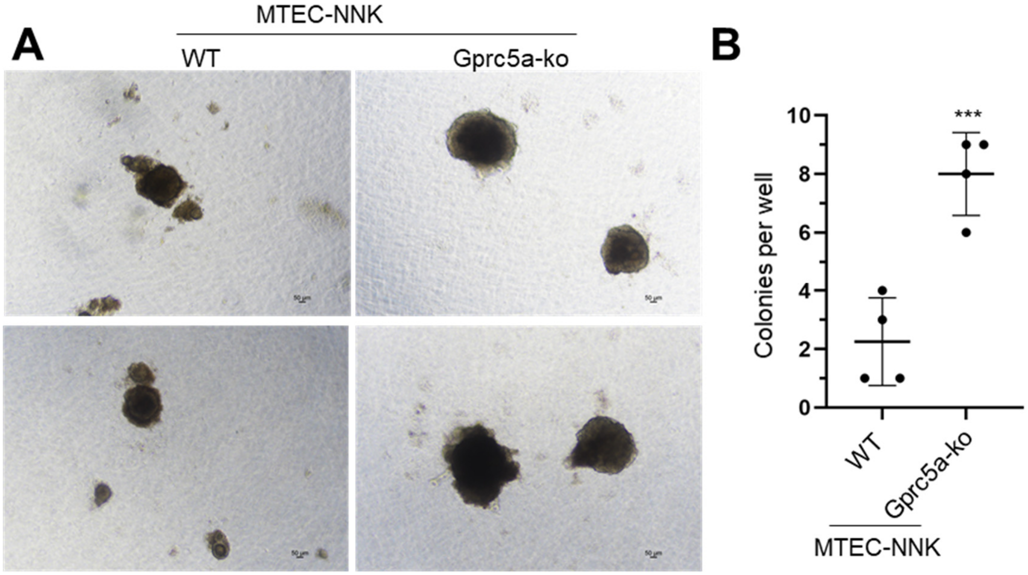

Supplementary Figure 7

NNK-treated Gprc5a-ko MTEC cells have stronger colony-formation capability than NNK-treated wild type METC cells in soft-agar assay. Wild type (WT) and Gprc5a-ko (KO) MTEC cells were repeatedly treated with NNK (100 pM) for 10 passages, leading to MTEC-KO-NNK10 and MTEC-WT-NNK10 cells. Five hundred cells per well (12 well plate) were seeded in soft agar with quadruplicates. After 3 weeks, the numbers and sizes of colonies were measured under microscope. *p<0.05; **p<0.01; ***p<0.001.

Supplementary Table 1. The primer sequence used in quantity real time PCR

Gene	Forward primer	Reverse primer
mGprc5a	ACCACAGACTTTGTGACCTGG	CGAGTGCAAACATGCAAGCC
mAtcb	GGCTGTATTCCCCTCCATCG	CCAGTTGGTAACAATGCCATGT
hGPRC5A	CTCACTCTCCCGATCCTCGT	CAGTCCGATGATGAAGGCGAA
hRelA	ATGTGGAGATCATTGAGCAGC	CCTGGTCCTGTGTAGCCATT
hcIAP2	TTTCCGTGGCTCTTATTCAAACT	GCACAGTGGTAGGAACTTCTCAT
hRARβ	TCCGAAAAGCTCACCAGGAAA	GGCCAGTTCACTGAATTTGTCC
hGAPDH	TGCACCACCAACTGCTTAGC	GGCATGGACTGTGGTCATGAG

Supplementary Table 2. The primer sequence used in ChIP assay

Gene	Forward primer	Reverse primer
hGPRC5A	TGGAACTGGAATAGGCGTGT	GTGAACTTGGTGCTGCTTCC
hGAPDH	TACTAGCGGTTTTACGGGCG	TCGAACAGGAGGAGCAGAGAGCGA
hIKBα	GACGACCCCAATTCAAATCG	TCAGGCTCGGGGAATTTCC

Uncropped/unedited gel document

Song et al (Submission to *JCI Insight*, August 2022)

NF-κB Represses Retinoic Acid Receptor-Mediated Transactivation of GPRC5A in Lung Epithelial Cells for Neoplasia

Note: This project was started from 2007 in Dr. Reuben Lotan's laboratory at the University of Texas MD Anderson Cancer Center. It was carried out by Dr. Xiaofeng Ye (co-first author of this manuscript) as part of his PhD thesis under the direct supervision of Dr. Jiong Deng (research assistant professor at that time). Both of them were working on the GPRC5A project at Dr. Lotan's group (please see reference below). Unfortunately, Dr. Lotan (1946-2011) passed away due to a terrible car accident in 2011, and his laboratory was closed 2-month later after this tragic accident. Dr. Ye graduated by summarizing his study/data on GPRC5A, including this uncompleted project, in his PhD thesis; he then performed a short period of post-doc study at Dr. Binhua P. Zhou's laboratory working on cancer metastasis. Dr. Deng went back to Shanghai Jiao-Tong University School of Medicine (2010-2021) to continue the GPCR5A study, including this project in his laboratory. The first author (Dr. Hongyong Song) is a post-doctoral fellow at Dr. Deng's group and he is the major driving force to finally complete this project. Due to the unexpected tragic accident of Dr. Lotan and the short notice of laboratory closure, Dr. Ye was unable to retrieve the original uncropped version of blots/films for **Fig 2D**, **3A**, **3B**, **5B**, **6C** and **6D** despite many efforts.

To resolve these issues, we have repeated these experiments (for Fig 2D, 3A, 3B, 5B, 6C, and 6D) in the last three months. We have obtained similar results. Now we have presented these uncropped/unedited images in this uncropped/unedited blot documents.

Reference:

Xiaofeng Ye, Qingguo Tao, Yafan Wang, Yijun Cheng, **Reuben Lotan**. Mechanisms underlying the induction of the putative human tumor suppressor GPRC5A by retinoic acid. Cancer Biol Ther. **2009** May;8(10):951-62.

Qingguo Tao, Junya Fujimoto, Taoyan Men, **Xiaofeng Ye**, **Jiong Deng**, Ludovic Lacroix, John L Clifford, Li Mao, Carolyn S Van Pelt, J Jack Lee, Dafna Lotan, **Reuben Lotan**. Identification of the retinoic acid-inducible Gprc5a as a new lung tumor suppressor gene. J Natl Cancer Inst. **2007** Nov 21;99(22):1668-82.

Jiong Deng, Junya Fujimoto, **Xiao-Feng Ye**, Tao-Yan Men, Carolyn S Van Pelt, Yu-Long Chen, Xiao-Feng Lin, Humam Kadara, Qingguo Tao, Dafna Lotan, **Reuben Lotan.** Knockout of the tumor suppressor gene Gprc5a in mice leads to NF-kappaB activation in airway epithelium and promotes lung inflammation and tumorigenesis. Cancer Prev Res (Phila). **2010** Apr;3(4):424-37.

Small Airway Epithelial Cells

Fig 2A

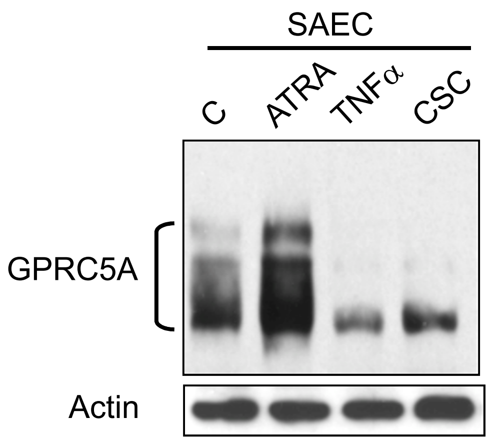

Fig 2B

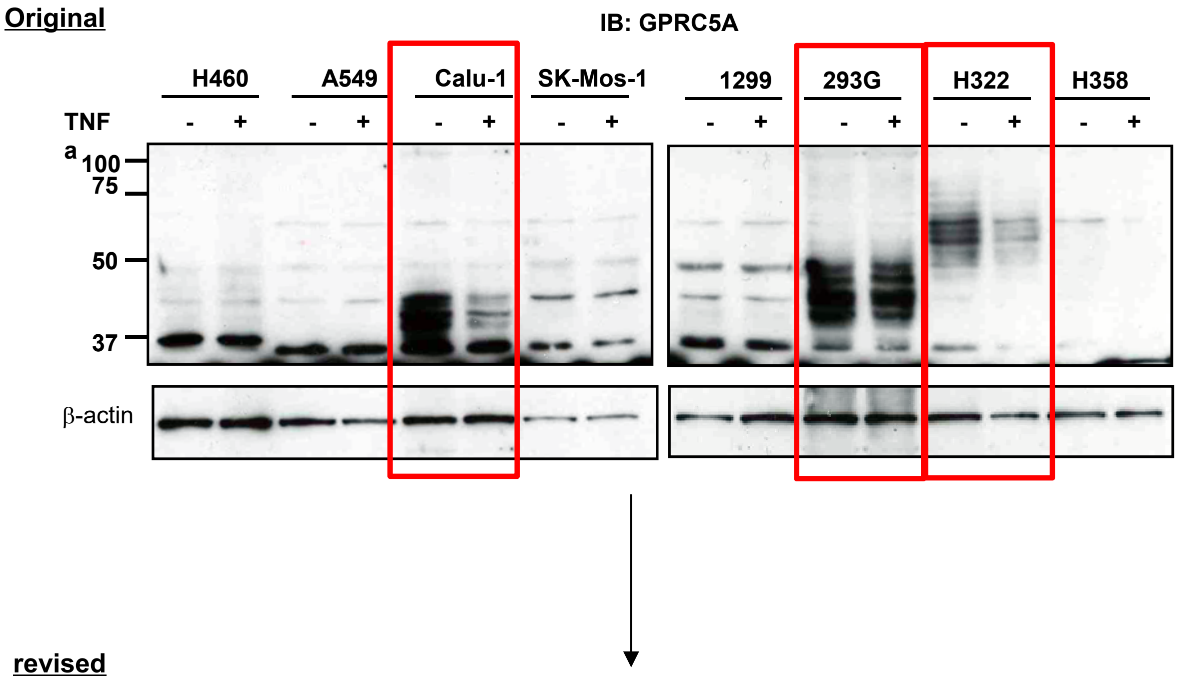

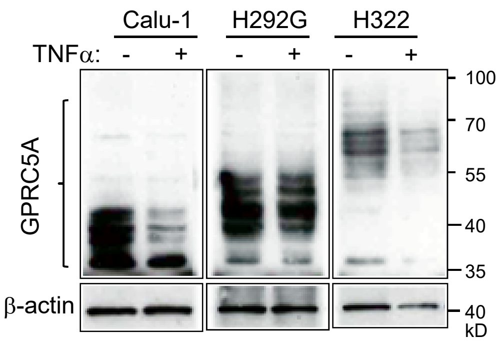

Fig 2F

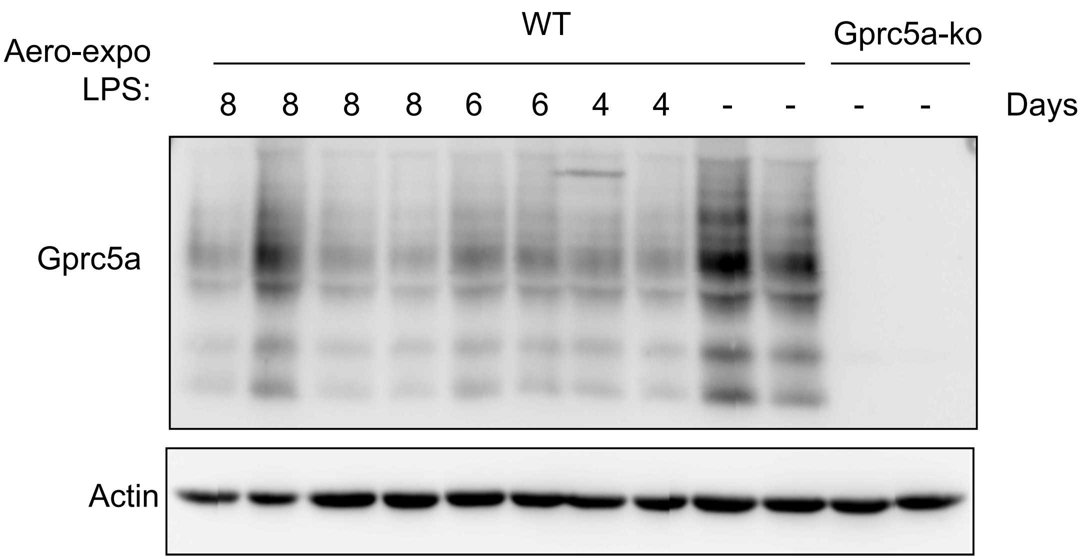

Fig 3D

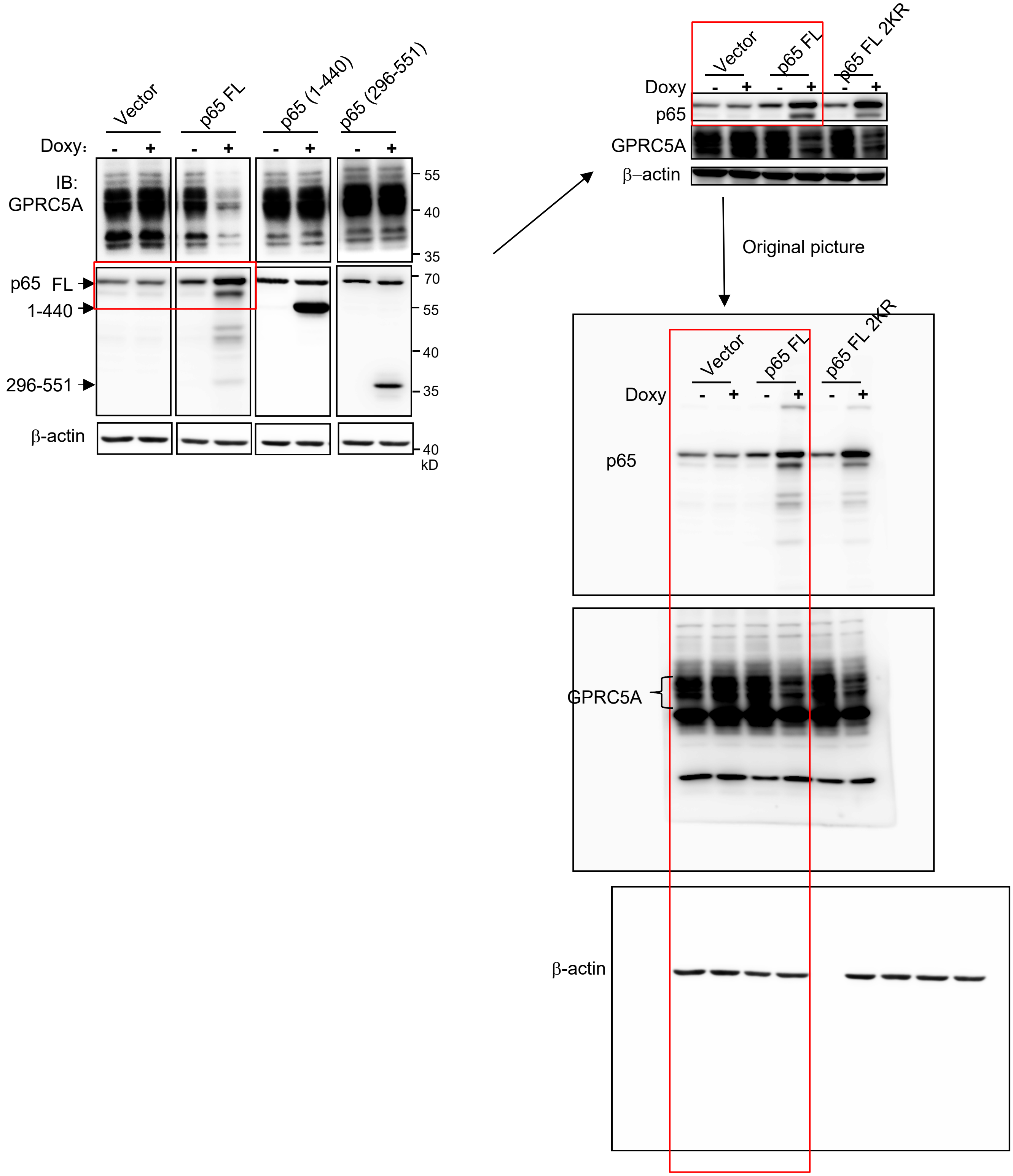

Vector
p65 FL
p65 (1-440)
p65 (296-551)
Doxy:
IB: GPRC5A
p65 FL
1-440
296-551
β-actin
55
40
35
70
55
40
35
40
kD
Vector
p65 FL
p65 FL 2KR
Doxy
p65
GPRC5A
β–actin
Original picture
Vector
p65 FL
p65 FL 2KR
Doxy
p65
GPRC5A
β-actin

Fig 3D

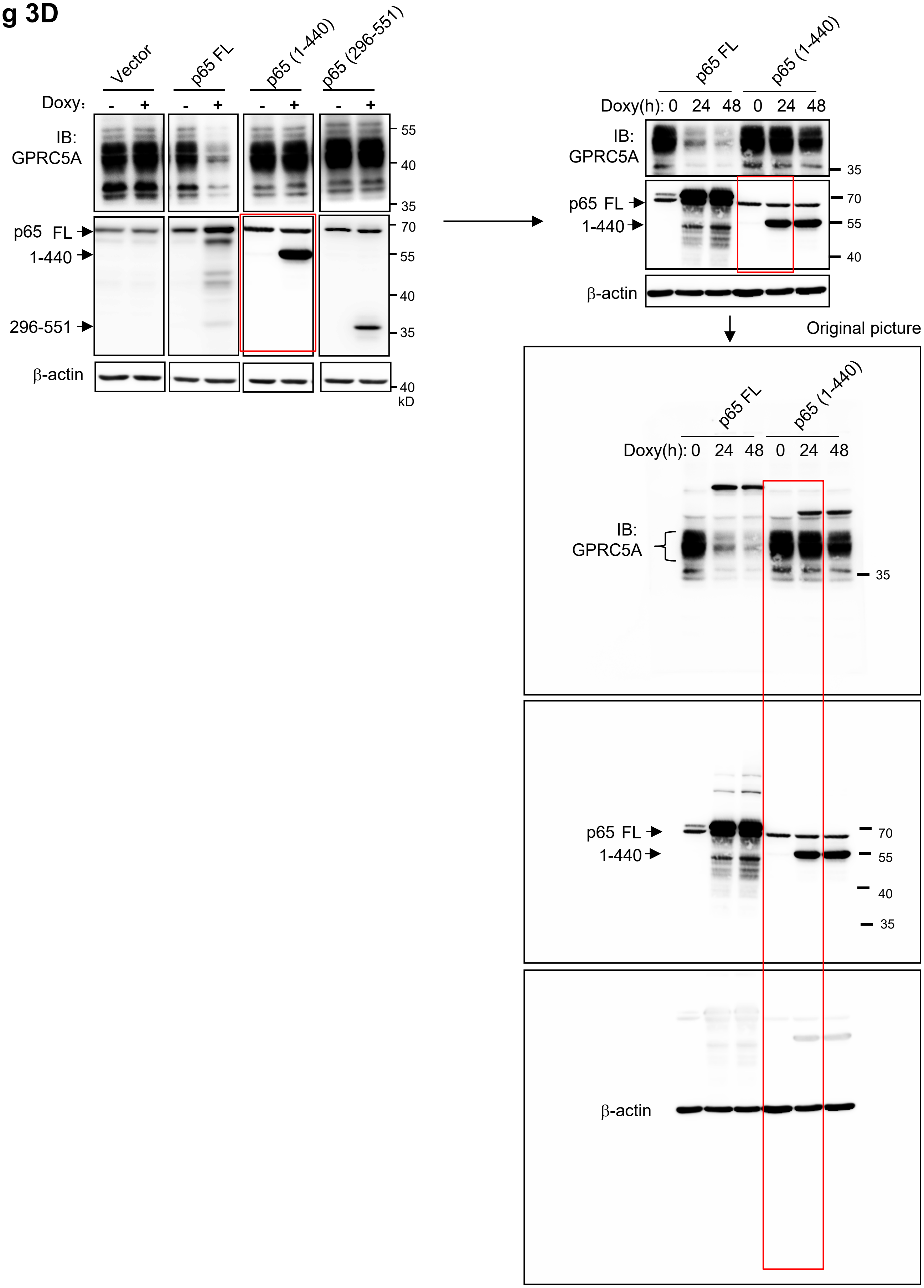

Vector
p65 FL
p65 (1-440)
p65 (296-551)
Doxy:
IB: GPRC5A
p65 FL
1-440
296-551
β-actin
55
40
35
70
55
40
35
40
kD
p65 FL
p65 (1-440)
Doxy(h): 0 24 48 0 24 48
IB: GPRC5A
p65 FL
1-440
β-actin
35
70
55
40
Original picture
p65 FL
p65 (1-440)
Doxy(h): 0 24 48 0 24 48
IB: GPRC5A
35
p65 FL
1-440
70
55
40
35
β-actin

Fig 3D

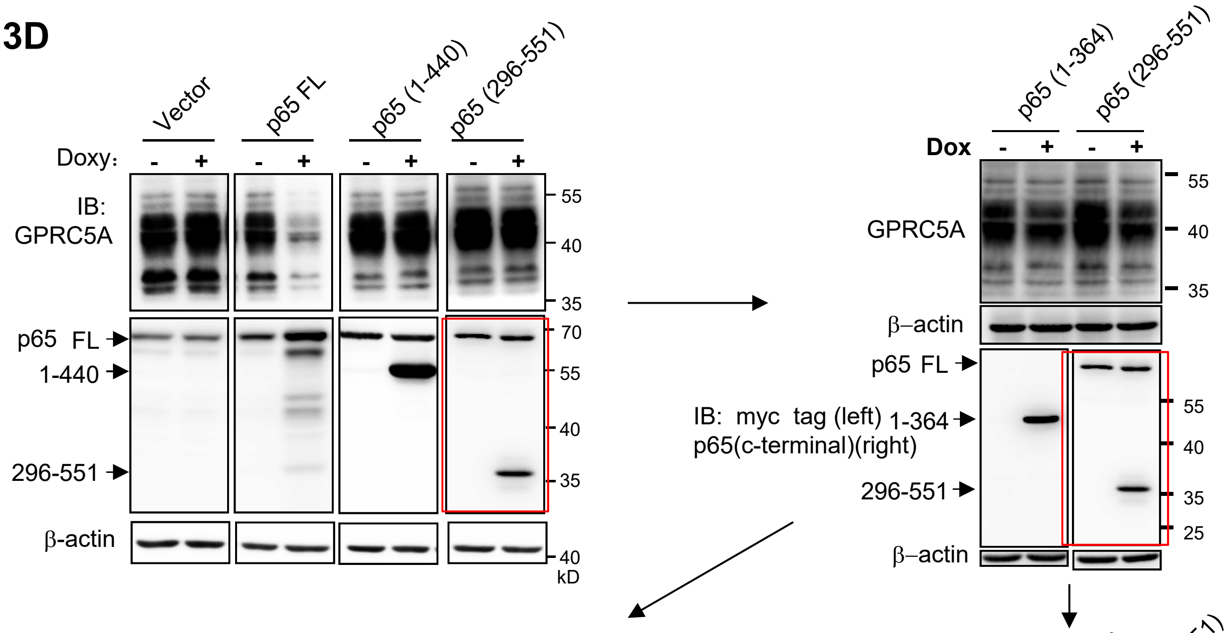

GPRC5A and p65 blot were run in the same gel, loading the same volume of sample.
And then were cropped to blot with different Ab.
P65(1-364) were blotted with myc-tag Ab, and because p65(296-551) without any tag.
so p65(296-551) were blotted with p65 Ab (target its c-terminal).
The following picture was the original experimental record for this blot.

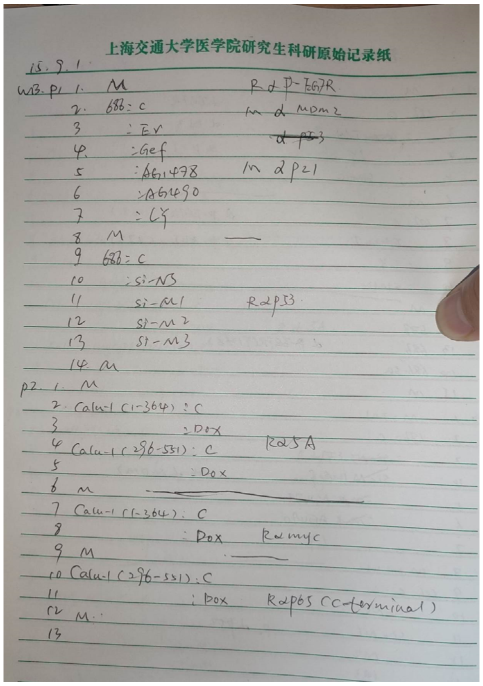
上海交通大学医学院研究生科研原始记录纸

15. 9. 1
WB. P1 1. M R α P-EGFR
2. 686: C M α MDM2
3 : Er ~~α p53~~
4. : Gef
5 : AG1478 M α p21
6 : AG490
7 : LY
8 M
9 686: C
10 : si-N3
11 si-M1 R α p53
12 si-M2
13 si-M3
14 M
p2. 1. M
2. Calu-1 (1-364): C
3 : Dox
4 Calu-1 (296-551): C R α 5A
5 : Dox
6 M
7 Calu-1 (1-364): C
8 : Dox R α myc
9 M
10 Calu-1 (296-551): C
11 : Dox R α p65 (C-terminal)
12 M.
13

Original picture

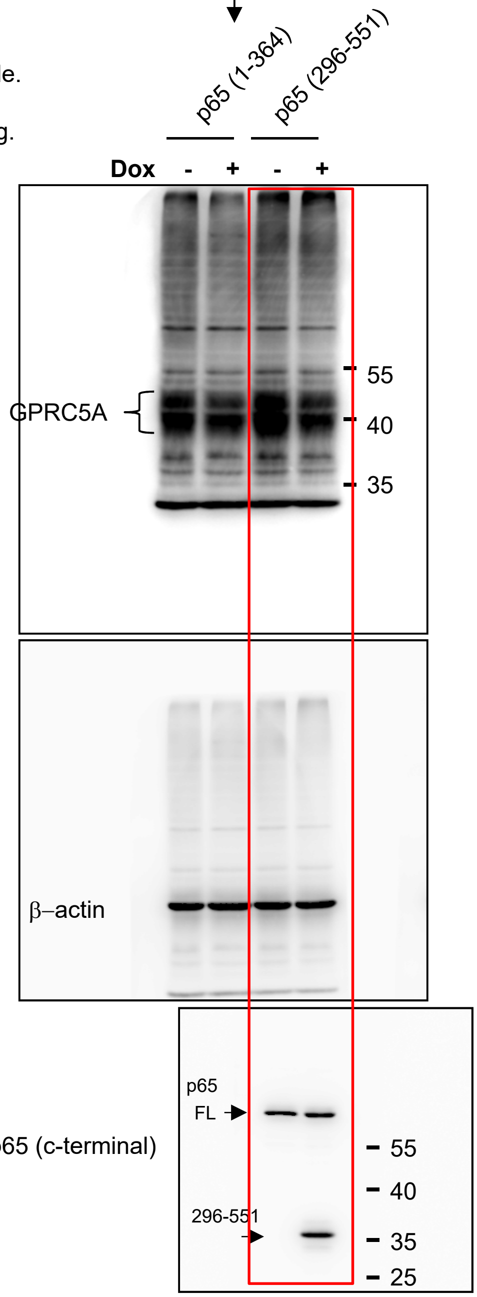

β–actin

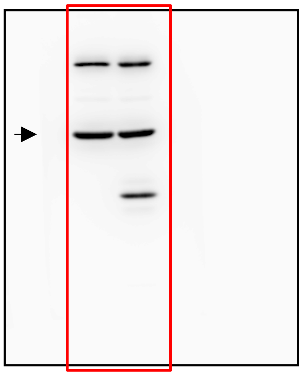

IB: p65 (c-terminal)

Fig 3E

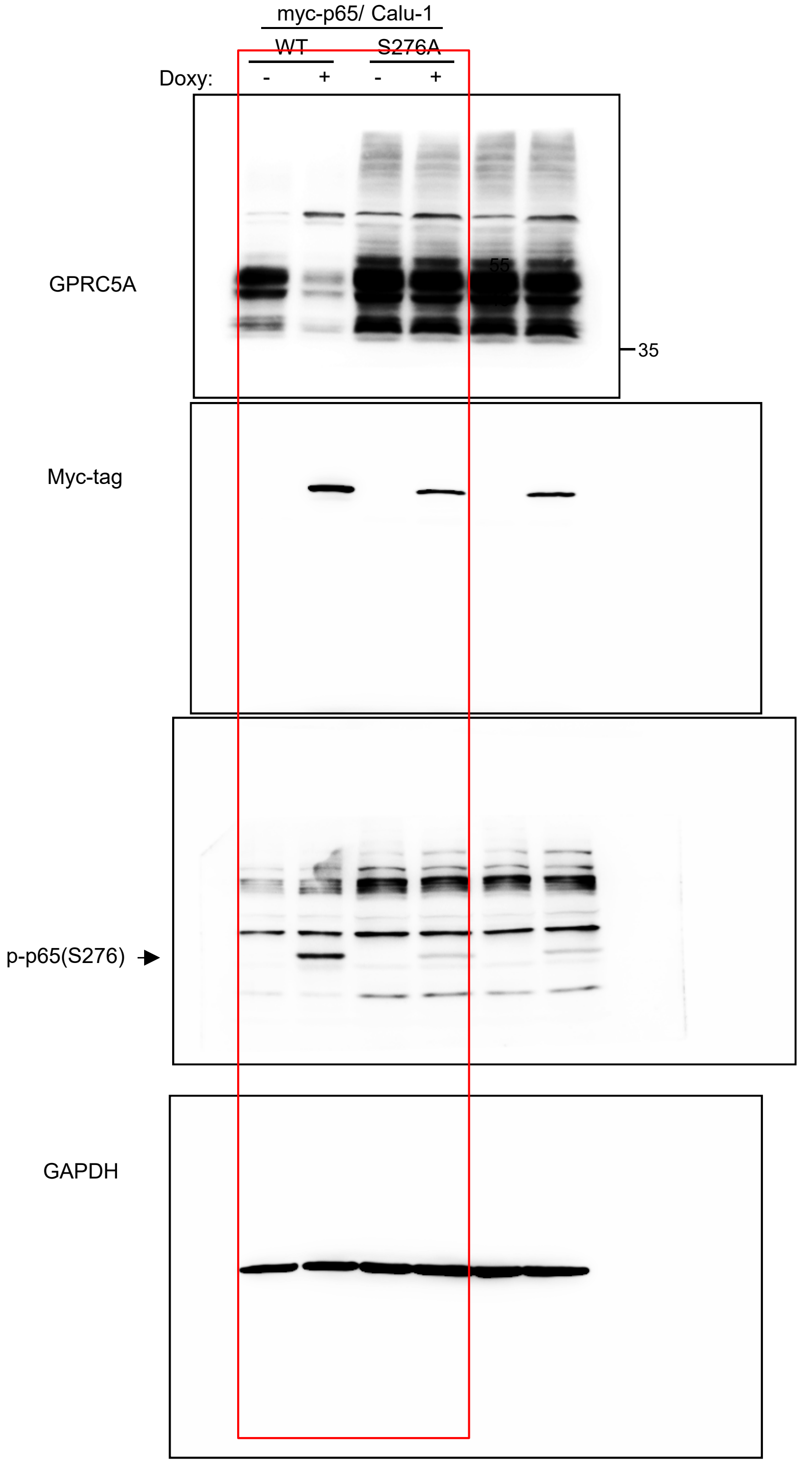
myc-p65/ Calu-1
WT
S276A
Doxy:
-
+
-
+
GPRC5A
55
35
Myc-tag
p-p65(S276)
GAPDH

Fig 4

A

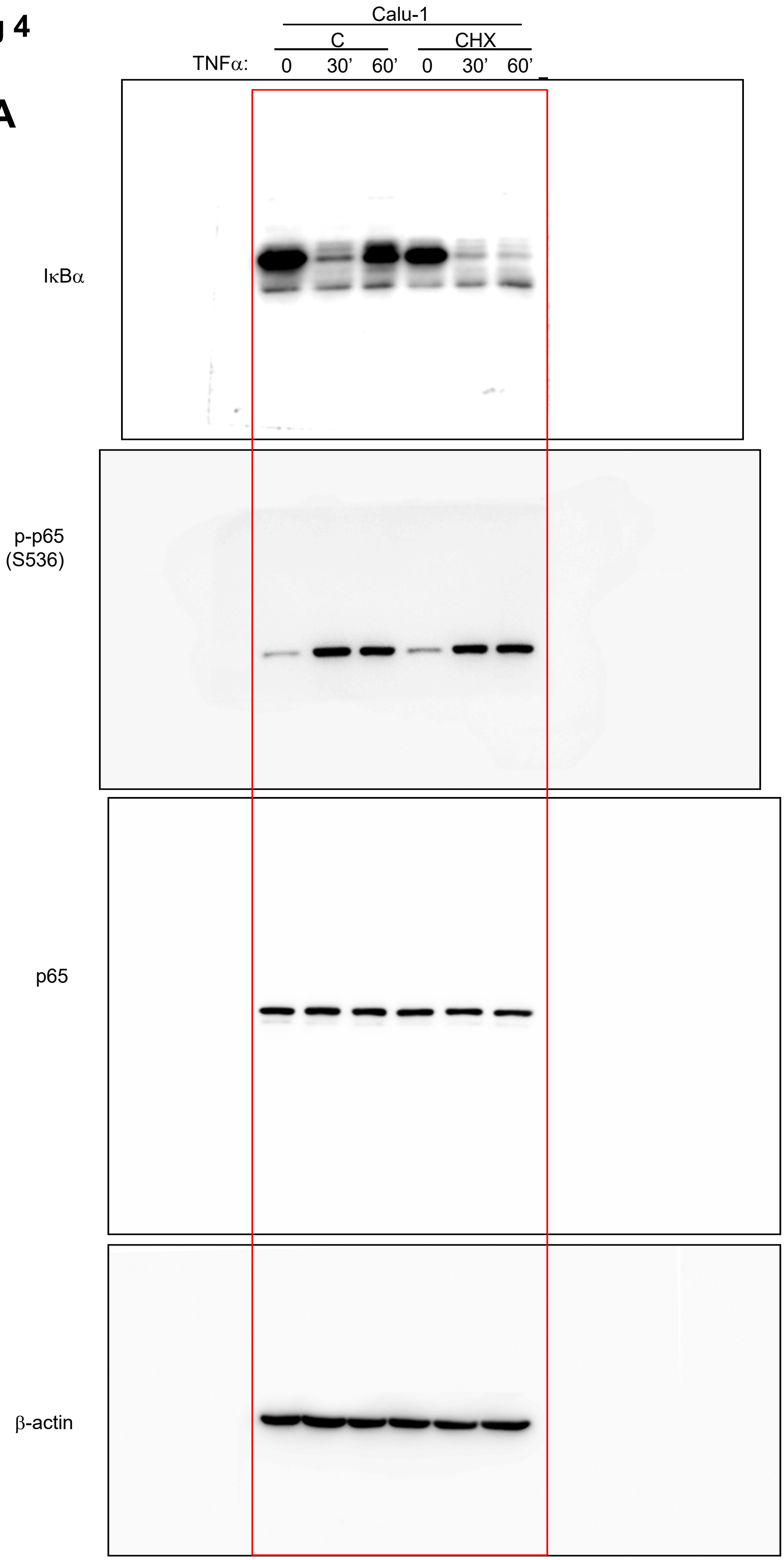

Fig 4

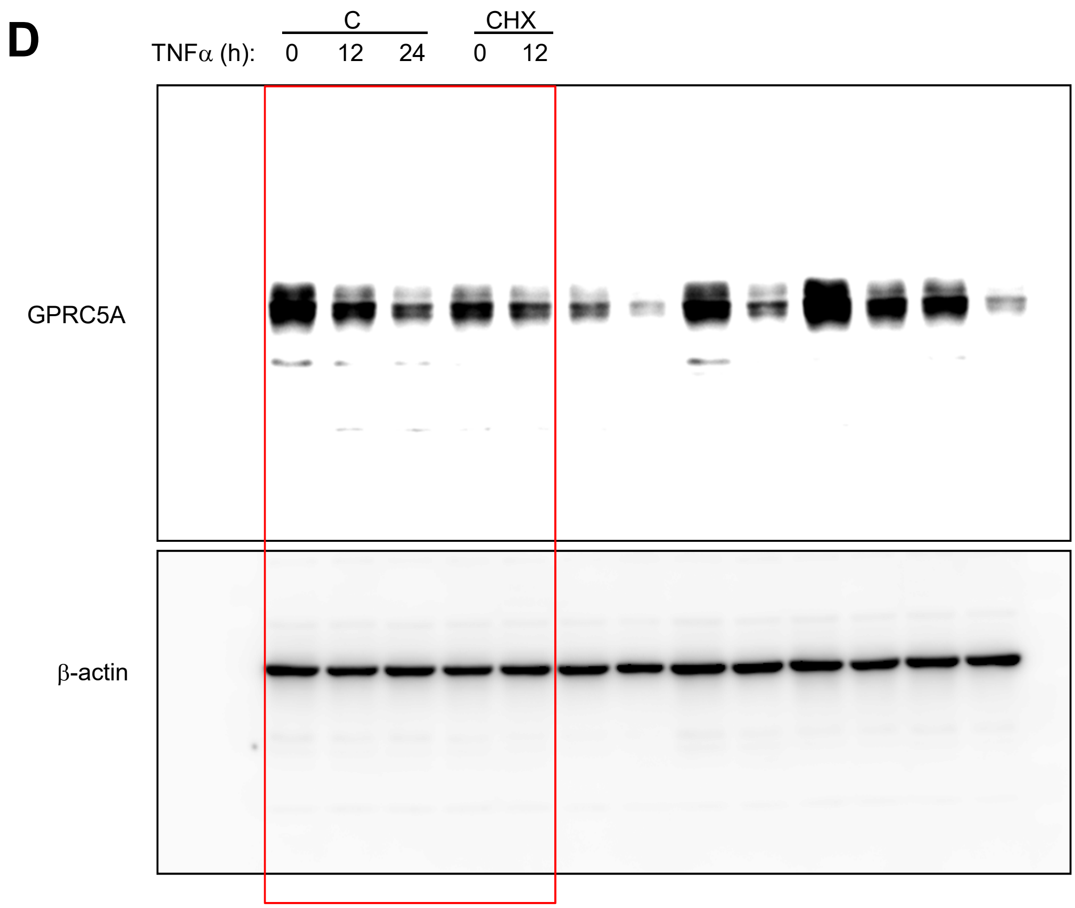

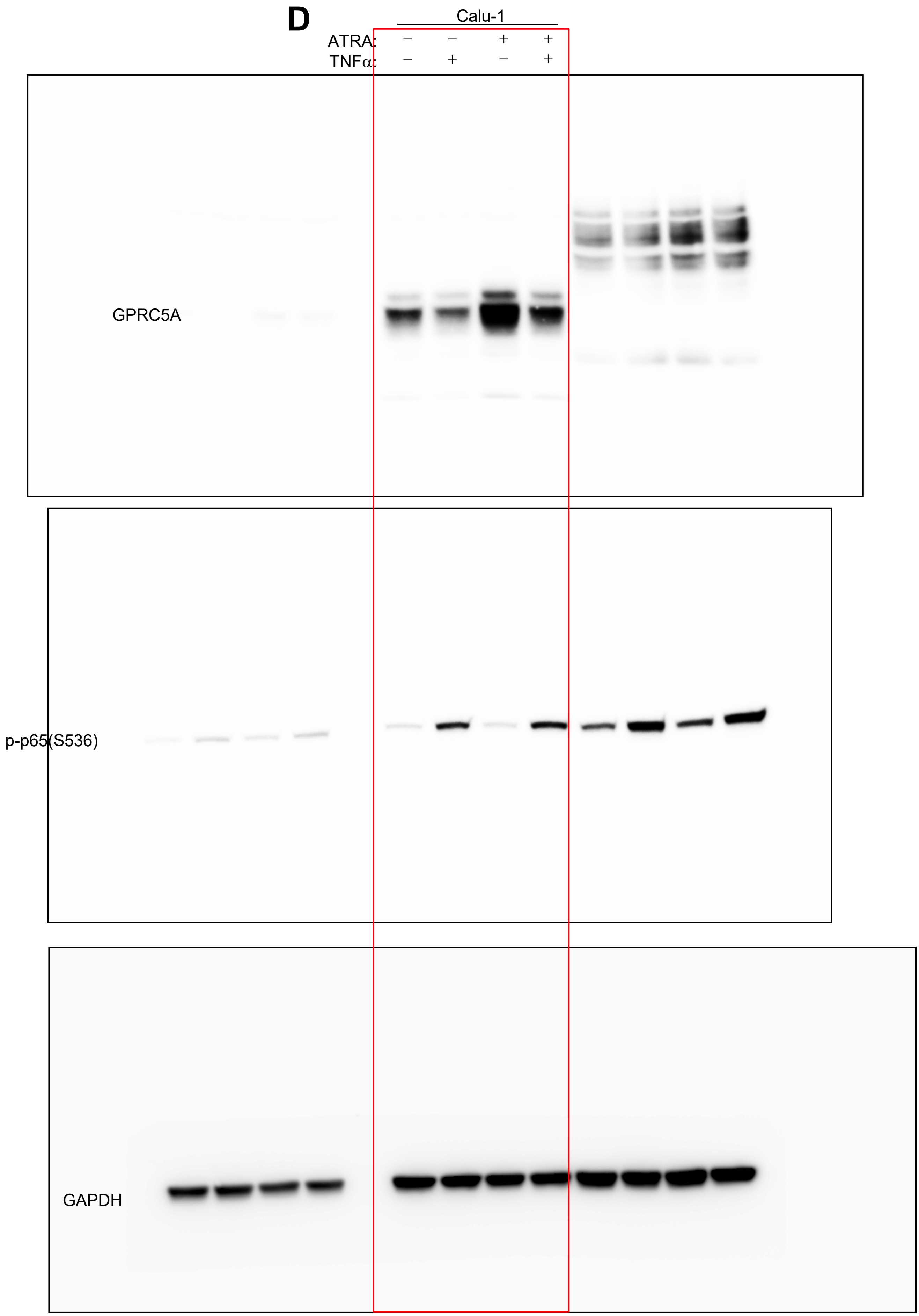
D
Calu-1
ATRA: − − + +
TNFα: − + − +
GPRC5A
p-p65(S536)
GAPDH

Fig 6 E

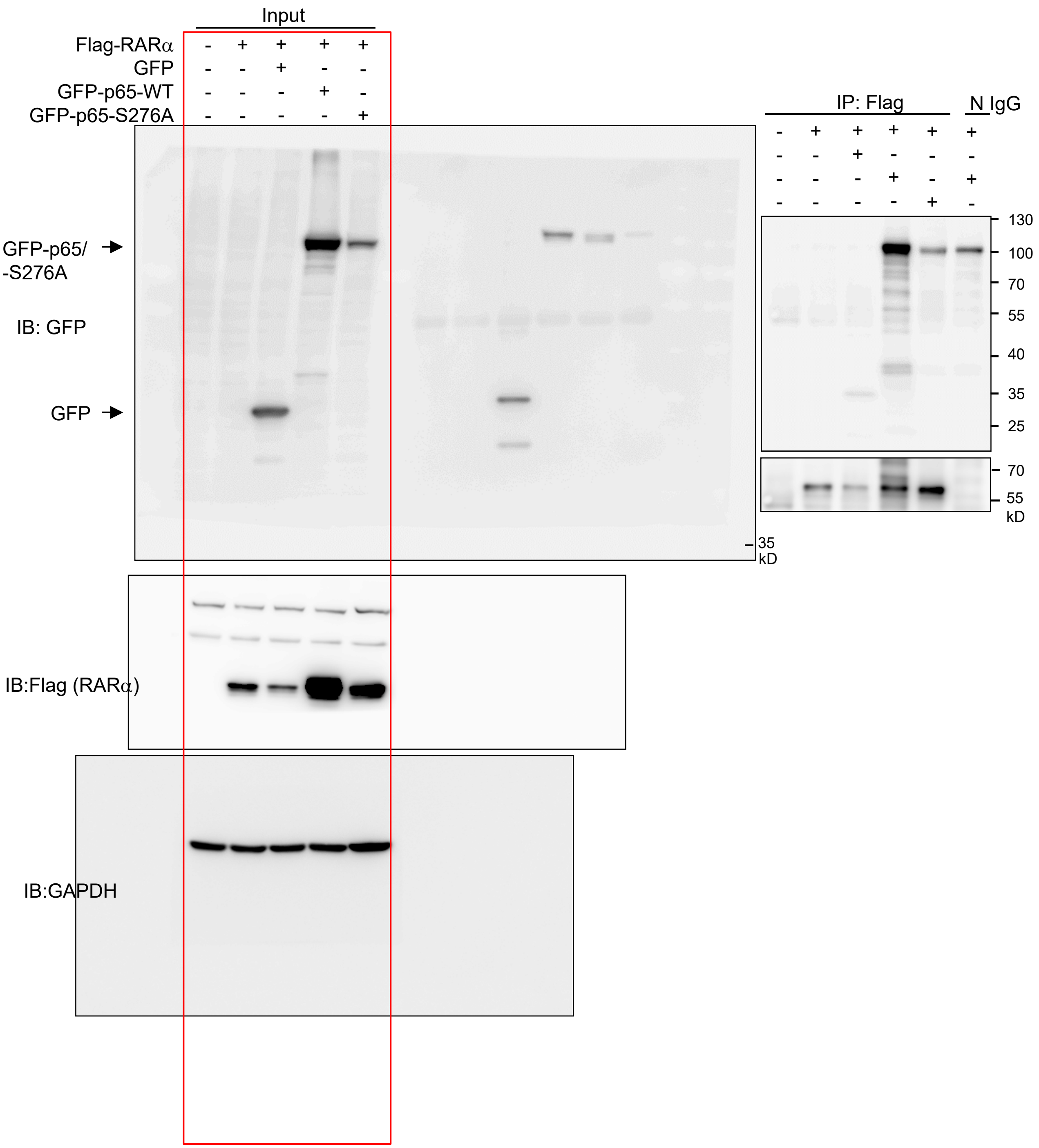
Input
Flag-RARα
GFP
GFP-p65-WT
GFP-p65-S276A
GFP-p65/
-S276A
IB: GFP
GFP
IB:Flag (RARα)
IB:GAPDH
IP: Flag
N IgG
130
100
70
55
40
35
25
70
55
kD
35
kD

Fig 6 E

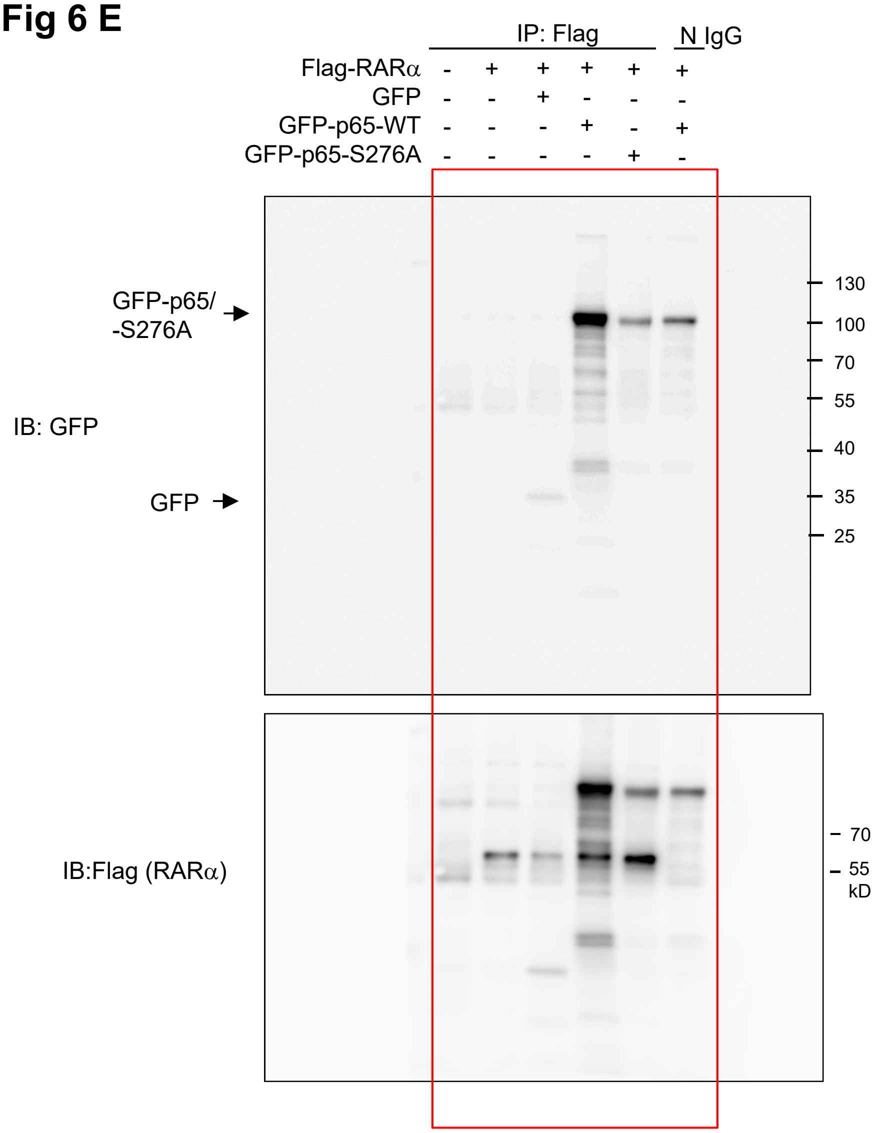

IP: Flag
N IgG
Flag-RARα - + + + + +
GFP - - + - - -
GFP-p65-WT - - - + - +
GFP-p65-S276A - - - - + -
GFP-p65/ -S276A
IB: GFP
GFP
130
100
70
55
40
35
25
IB:Flag (RARα)
70
55
kD

Fig 7

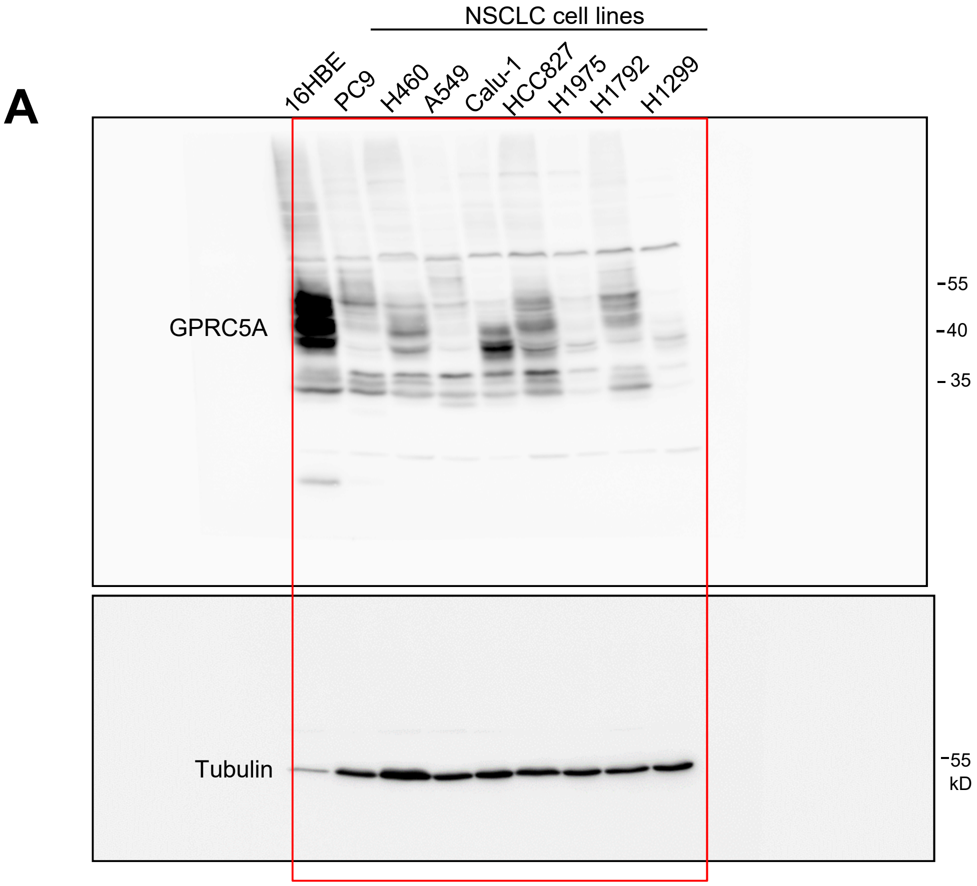
A
NSCLC cell lines
16HBE
PC9
H460
A549
Calu-1
HCC827
H1975
H1792
H1299
GPRC5A
55
40
35
Tubulin
55
kD

Fig 7

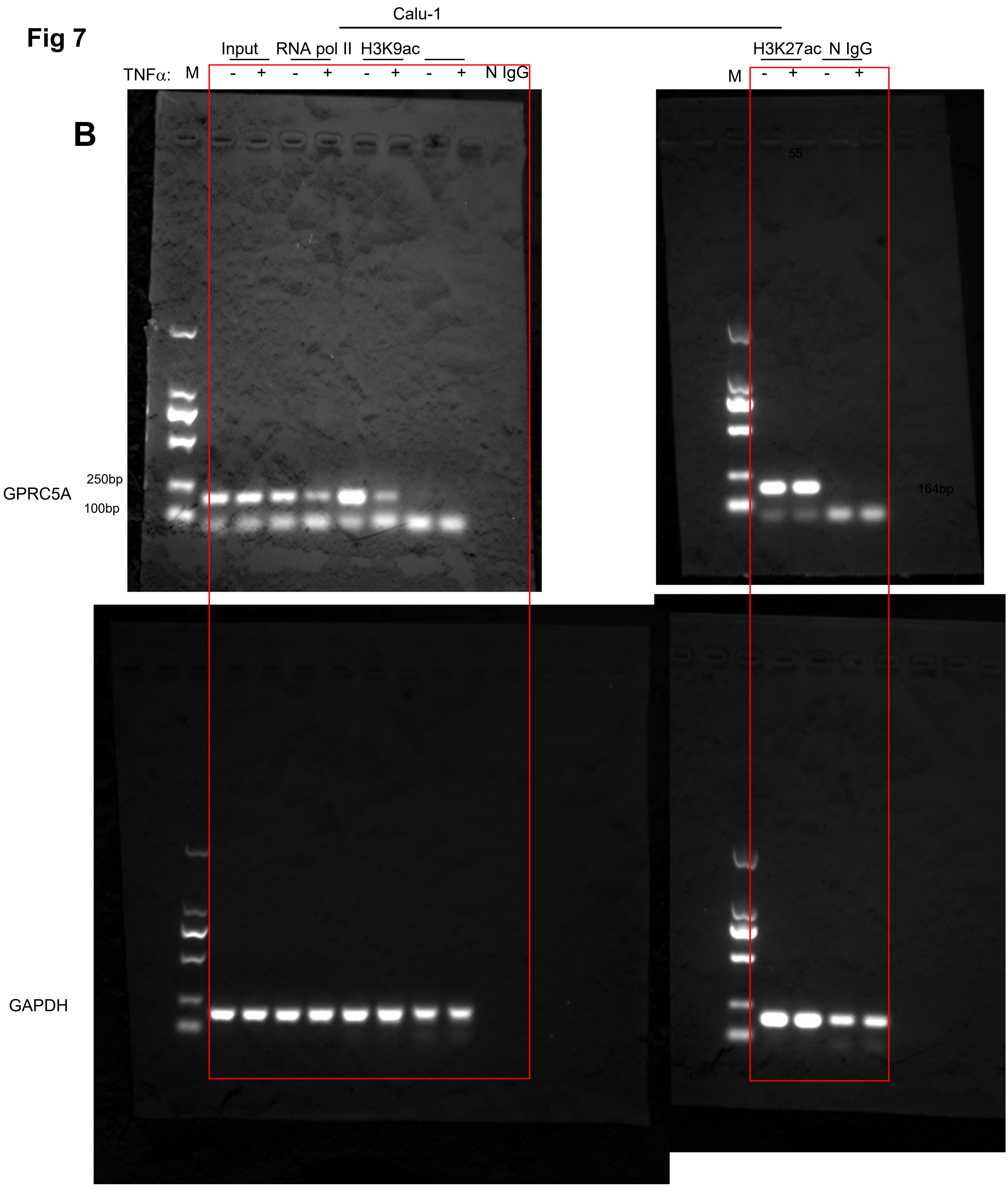
Calu-1
Input
RNA pol II
H3K9ac
H3K27ac
N IgG
TNFα:
M
- + - + - + - +
N IgG
B
GPRC5A
250bp
100bp
164bp
55
GAPDH

Fig 7

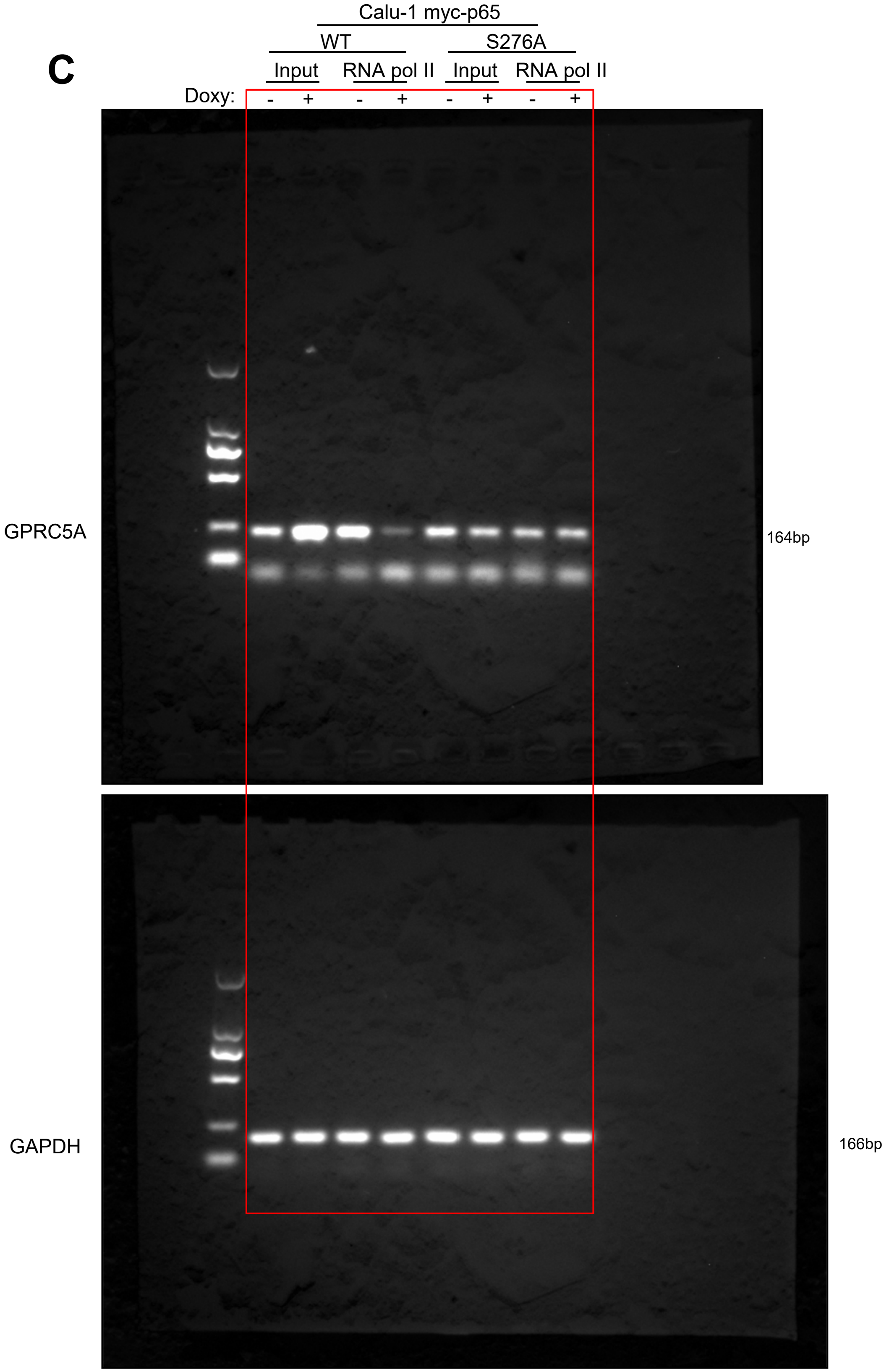
C
Calu-1 myc-p65
WT
S276A
Input
RNA pol II
Input
RNA pol II
Doxy: - + - + - + - +
GPRC5A
164bp
GAPDH
166bp

Fig 7

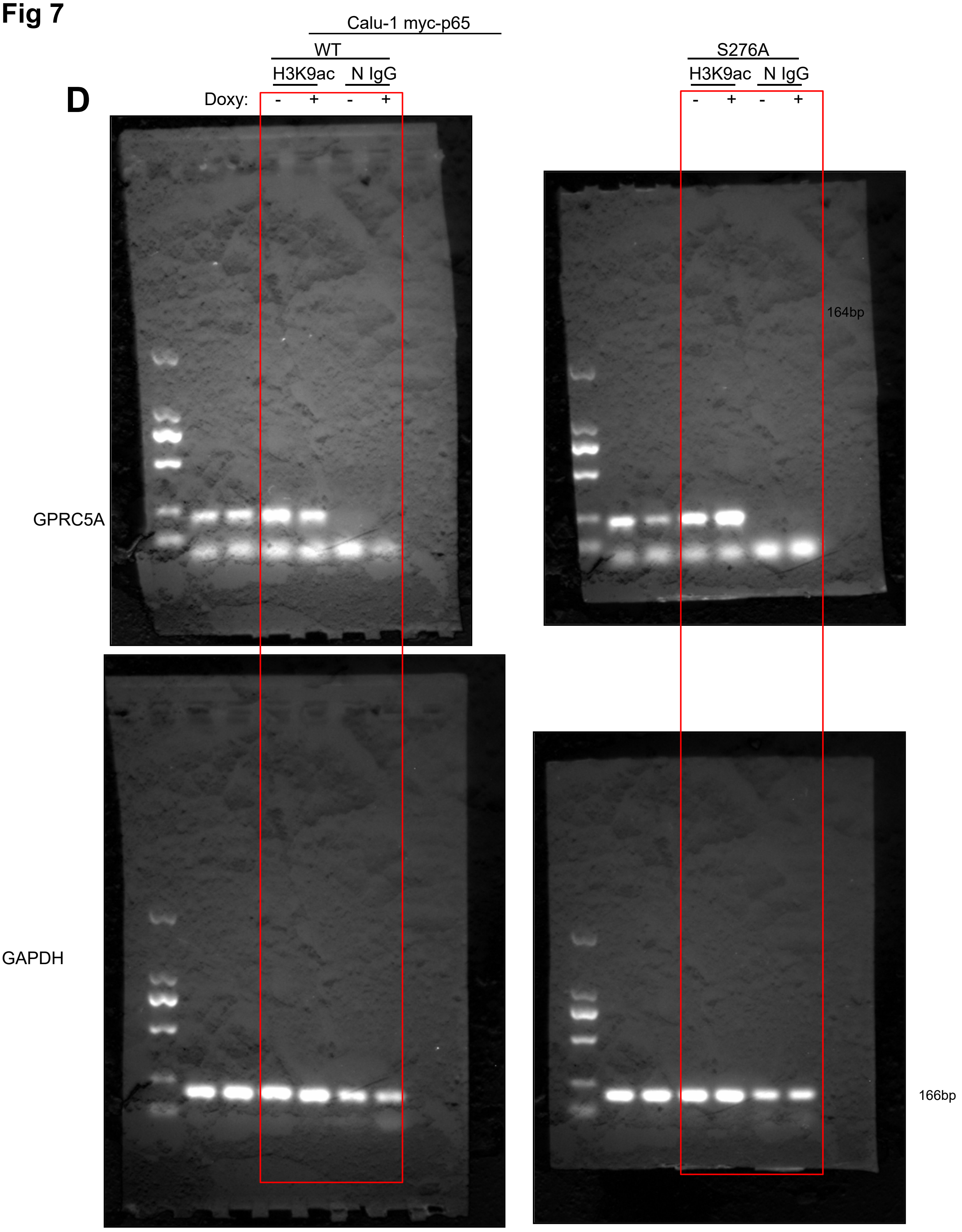
Calu-1 myc-p65
WT
H3K9ac
N IgG
S276A
H3K9ac
N IgG
D
Doxy:
-
+
-
+
-
+
-
+
GPRC5A
164bp
GAPDH
166bp

Fig 7

E

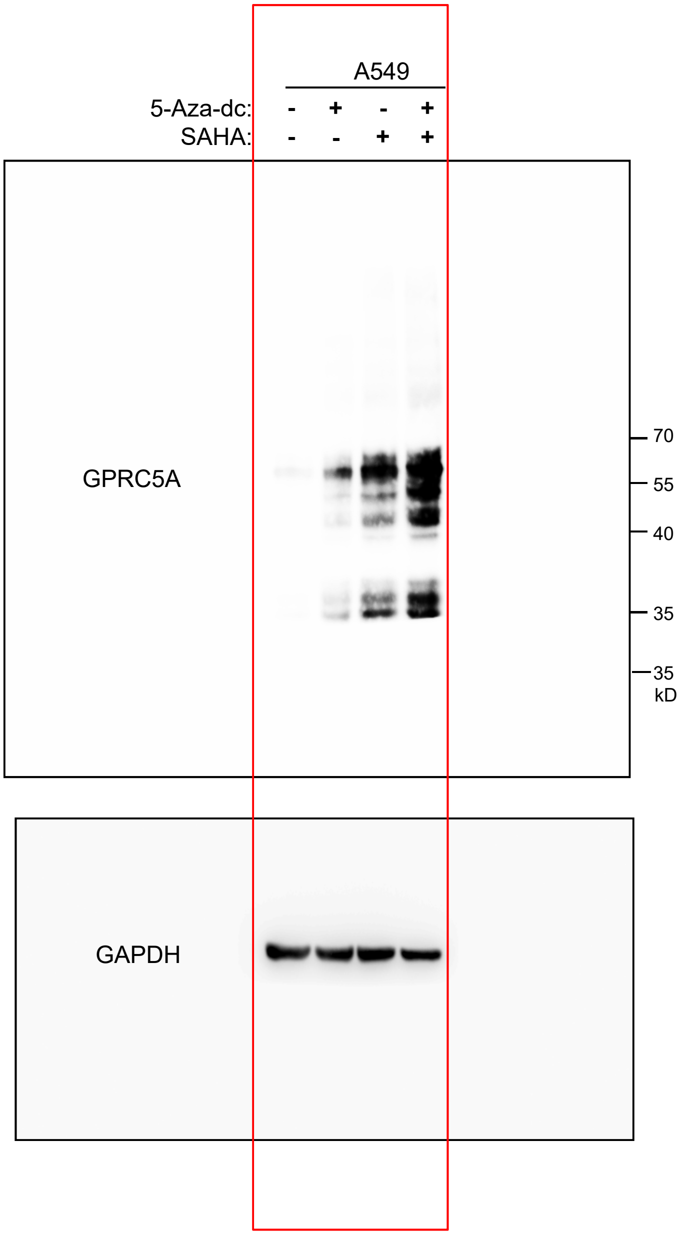

Fig 7

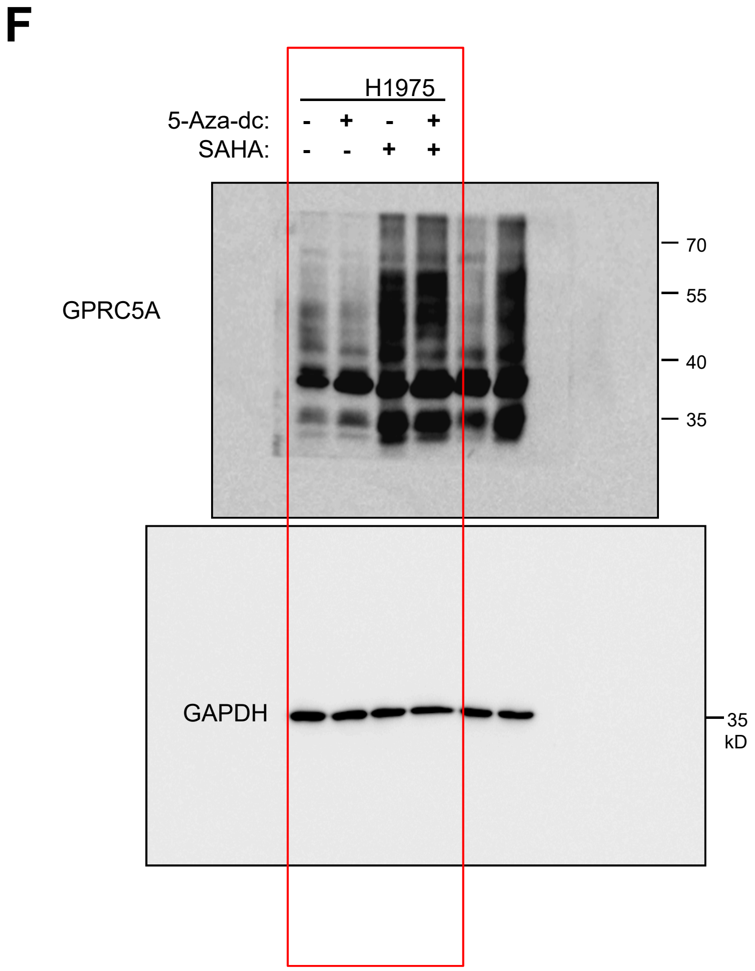

Fig 7

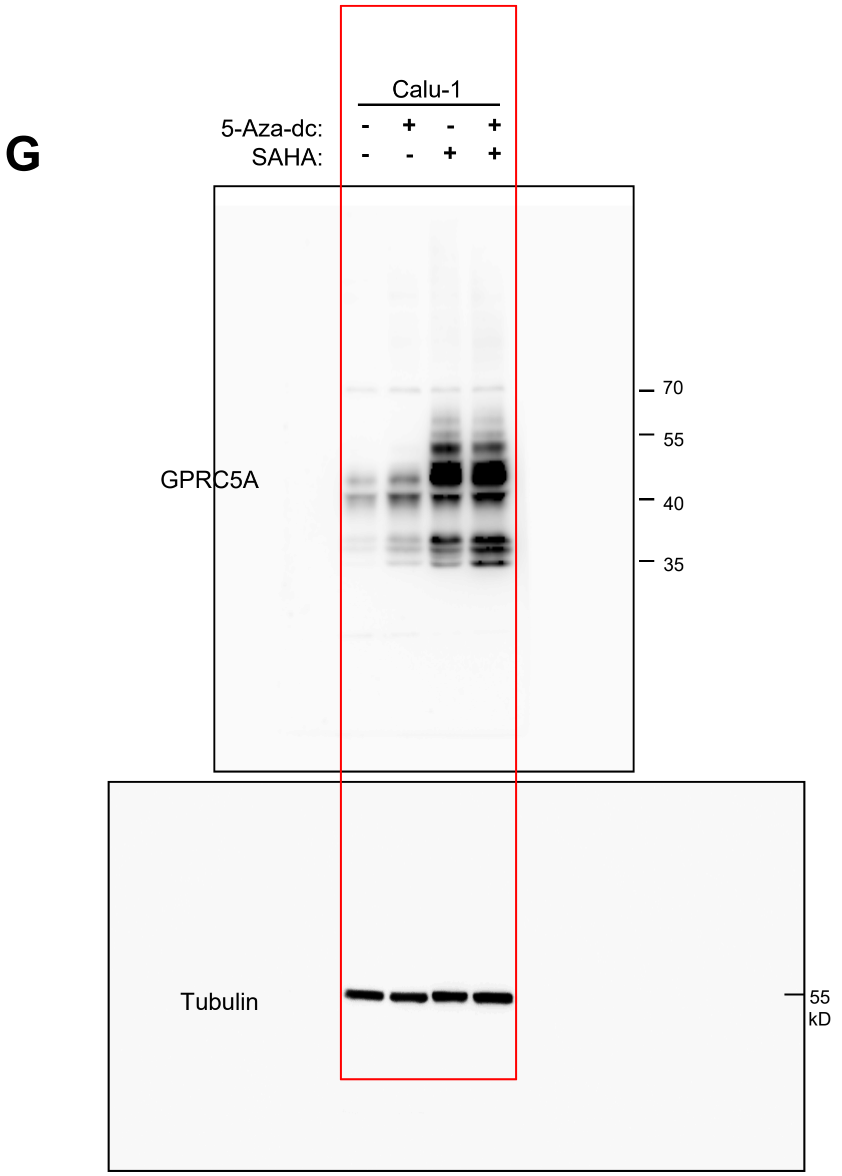

Fig 7

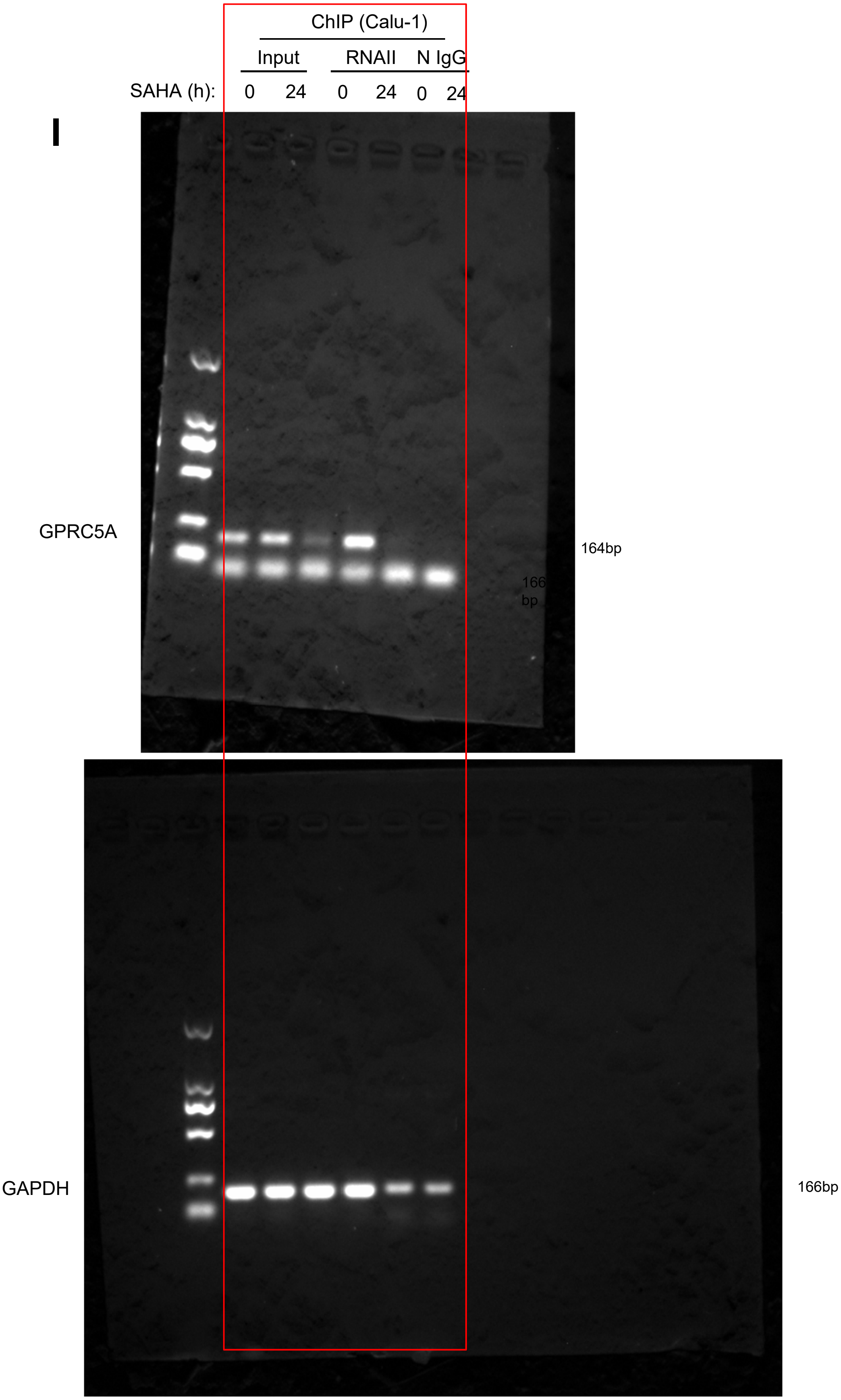
ChIP (Calu-1)
Input
RNAII
N IgG
SAHA (h):
0
24
0
24
0
24
I
GPRC5A
164bp
166
bp
GAPDH
166bp

Fig 7

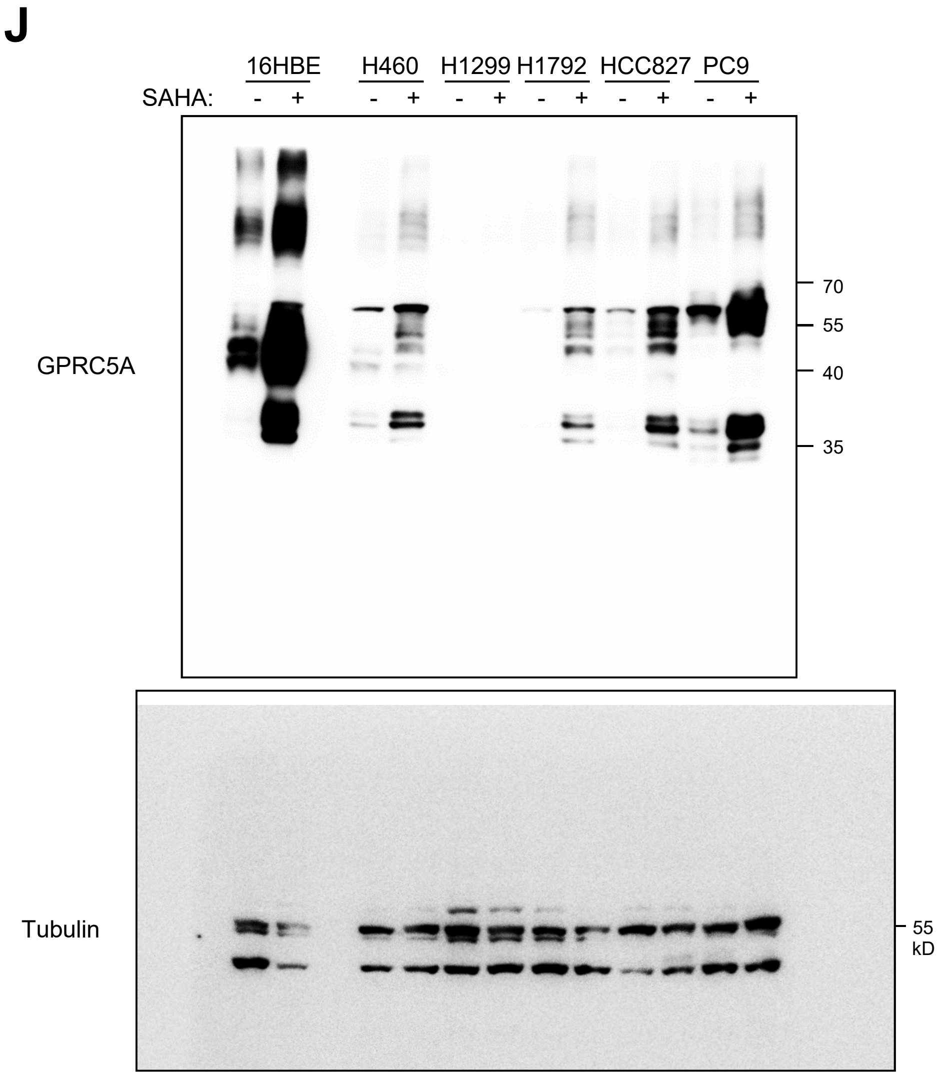
J
16HBE
H460
H1299
H1792
HCC827
PC9
SAHA:
-
+
-
+
-
+
-
+
-
+
-
+
GPRC5A
70
55
40
35
Tubulin
55
kD

Fig 7

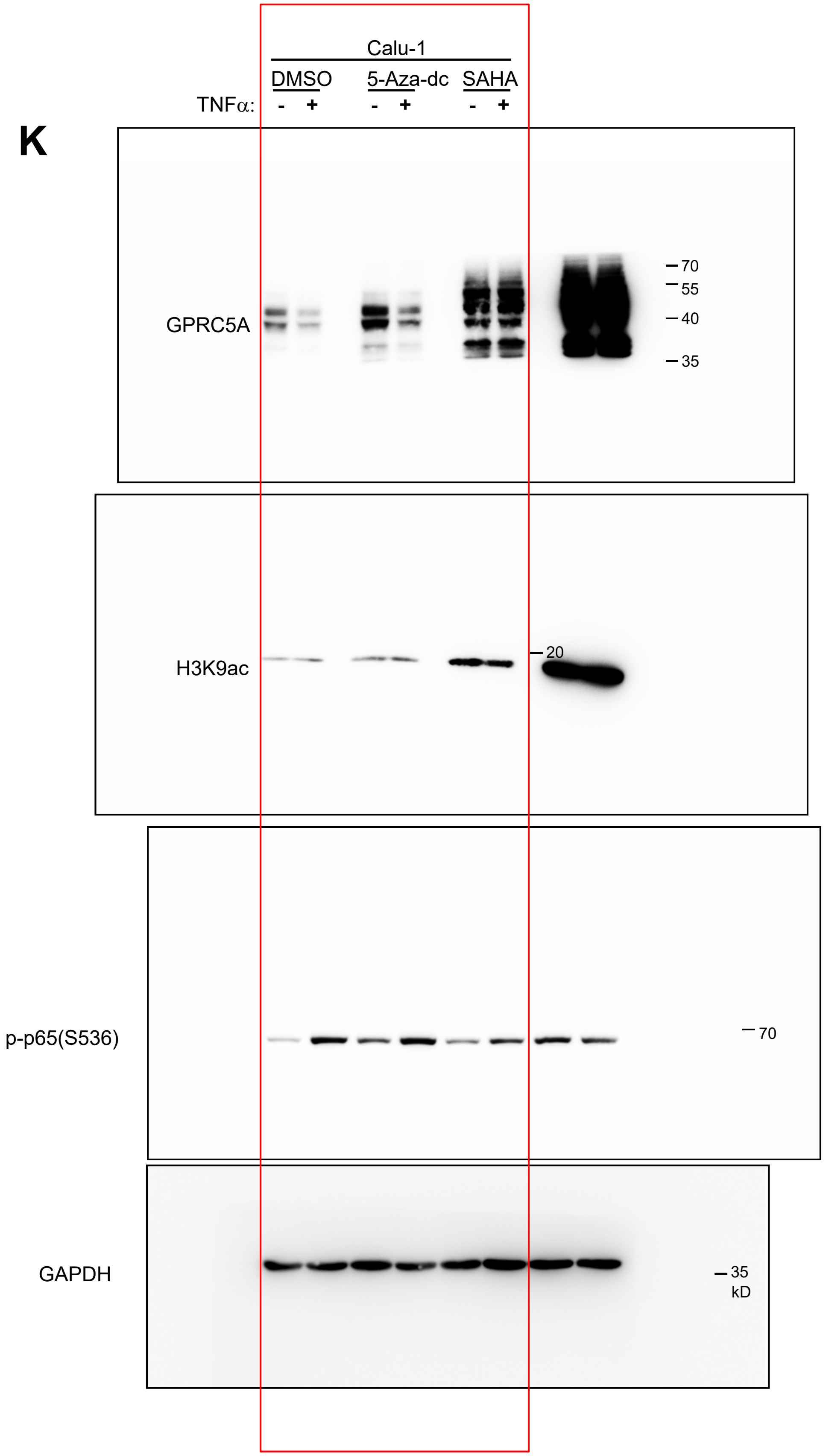
K
Calu-1
DMSO
5-Aza-dc
SAHA
TNFα:
- + - + - +
GPRC5A
70
55
40
35
H3K9ac
20
p-p65(S536)
70
GAPDH
35
kD

Figure 2D

Old data

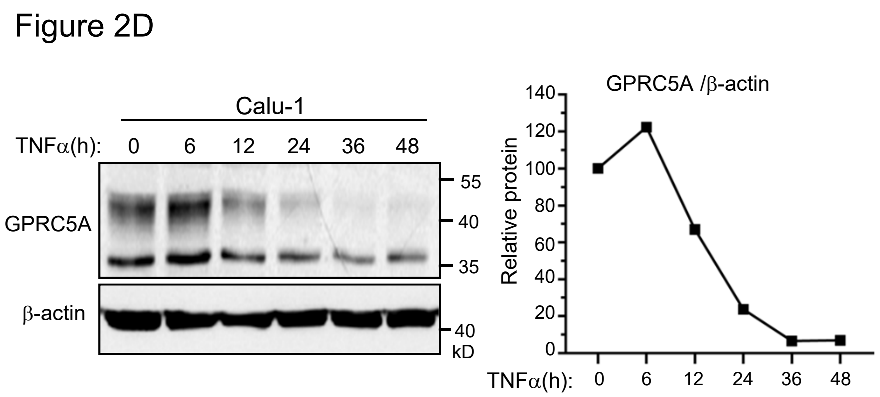

New data

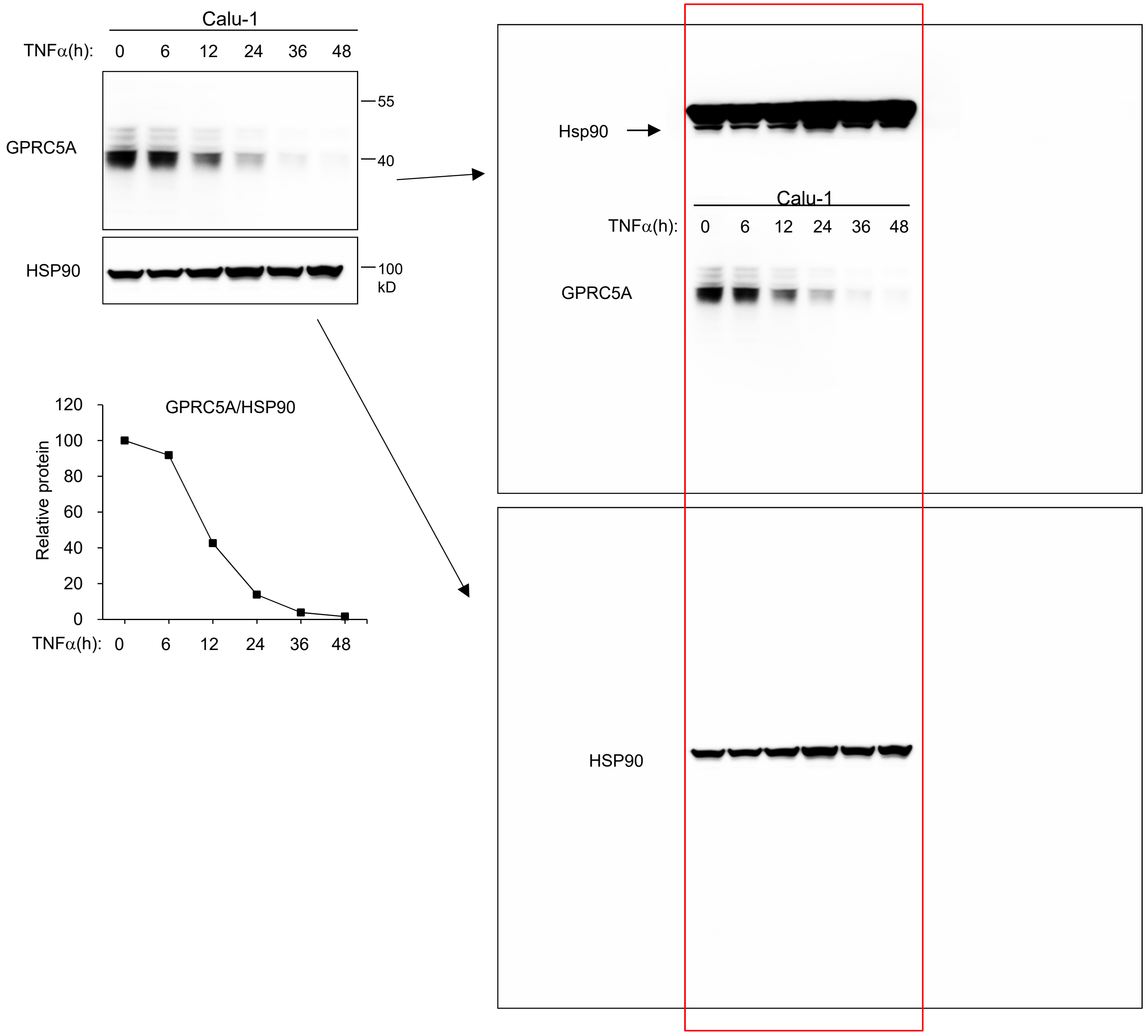

Figure 3A

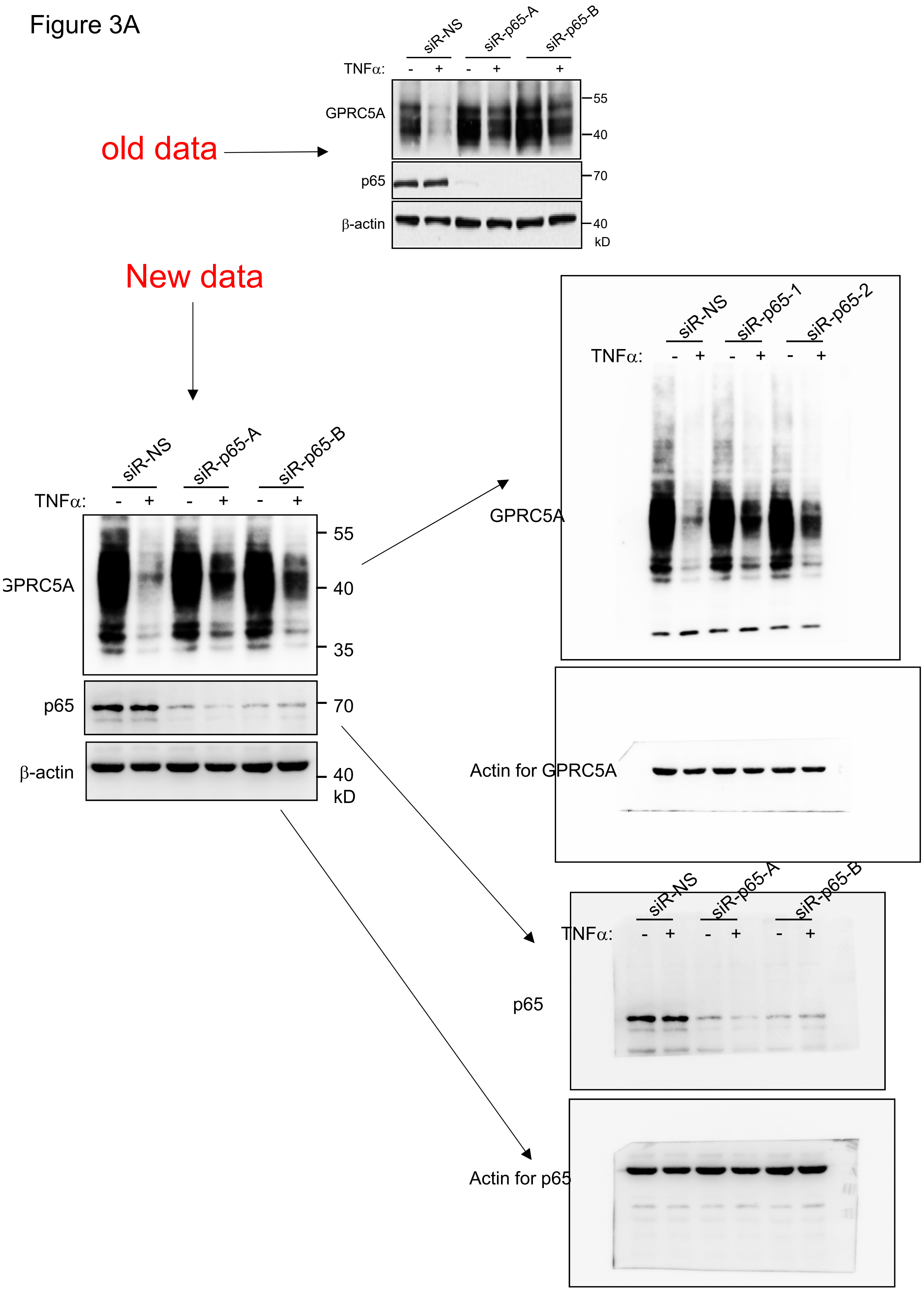
old data
siR-NS
siR-p65-A
siR-p65-B
TNFα: - + - + - +
GPRC5A
p65
β-actin
55
40
70
40
kD
New data
siR-NS
siR-p65-A
siR-p65-B
TNFα: - + - + - +
GPRC5A
p65
β-actin
55
40
35
70
40
kD
siR-NS
siR-p65-1
siR-p65-2
TNFα: - + - + - +
GPRC5A
Actin for GPRC5A
siR-NS
siR-p65-A
siR-p65-B
TNFα: - + - + - +
p65
Actin for p65

Figure 3B

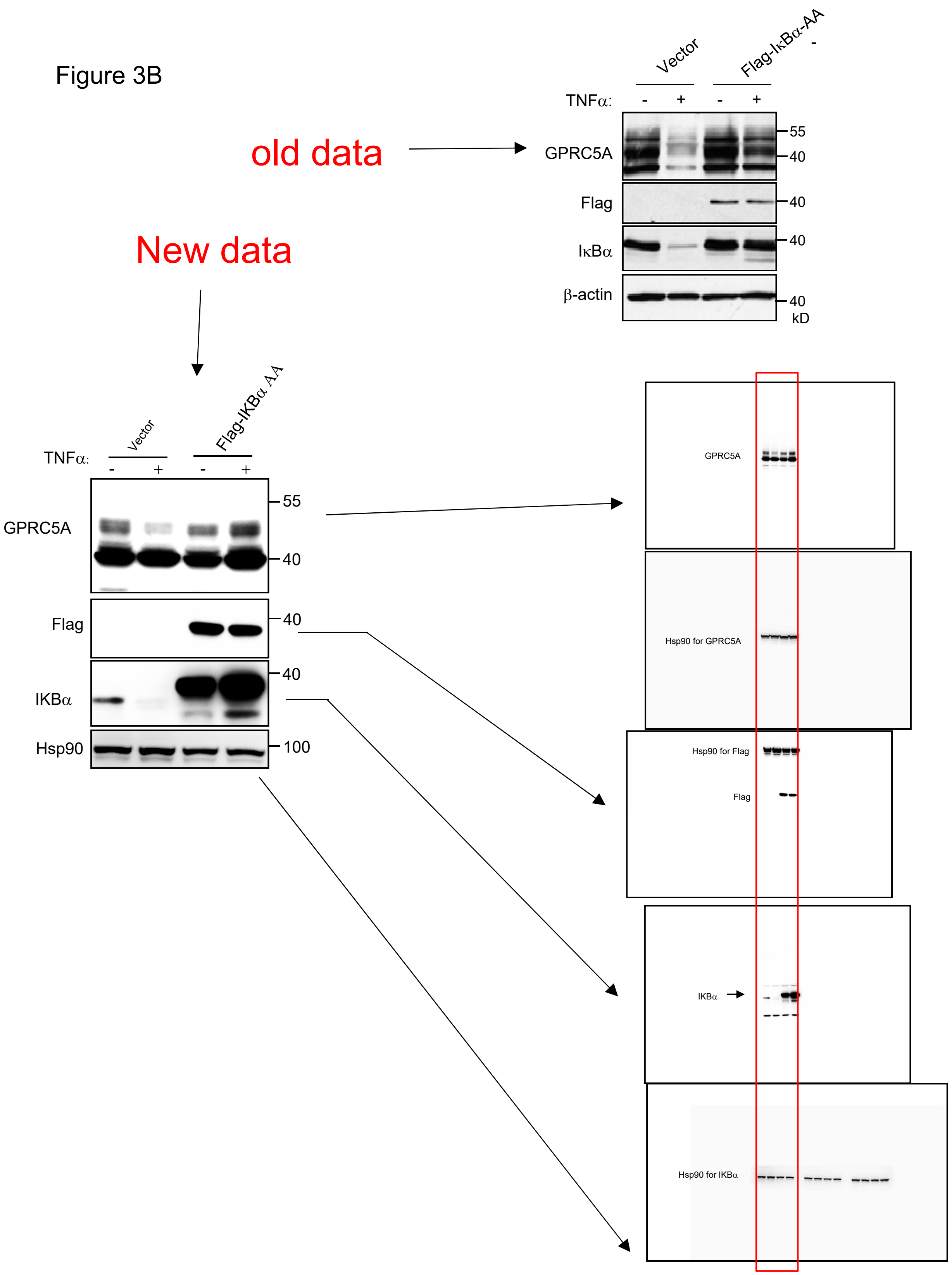
old data
New data
Vector
Flag-IκBα-AA
TNFα:
- + - +
GPRC5A
55
40
Flag
40
IκBα
40
β-actin
40
kD
Vector
Flag-IKBα AA
TNFα:
- + - +
GPRC5A
55
40
Flag
40
IKBα
40
Hsp90
100
GPRC5A
Hsp90 for GPRC5A
Hsp90 for Flag
Flag
IKBα
Hsp90 for IKBα

Figure 5

Old data

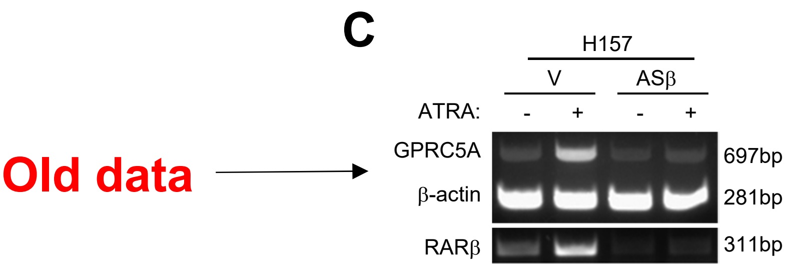

New data

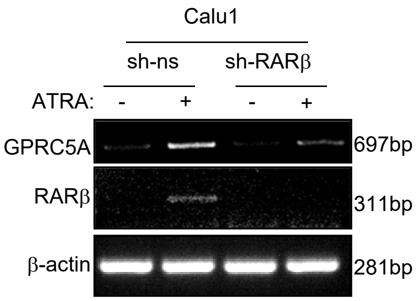

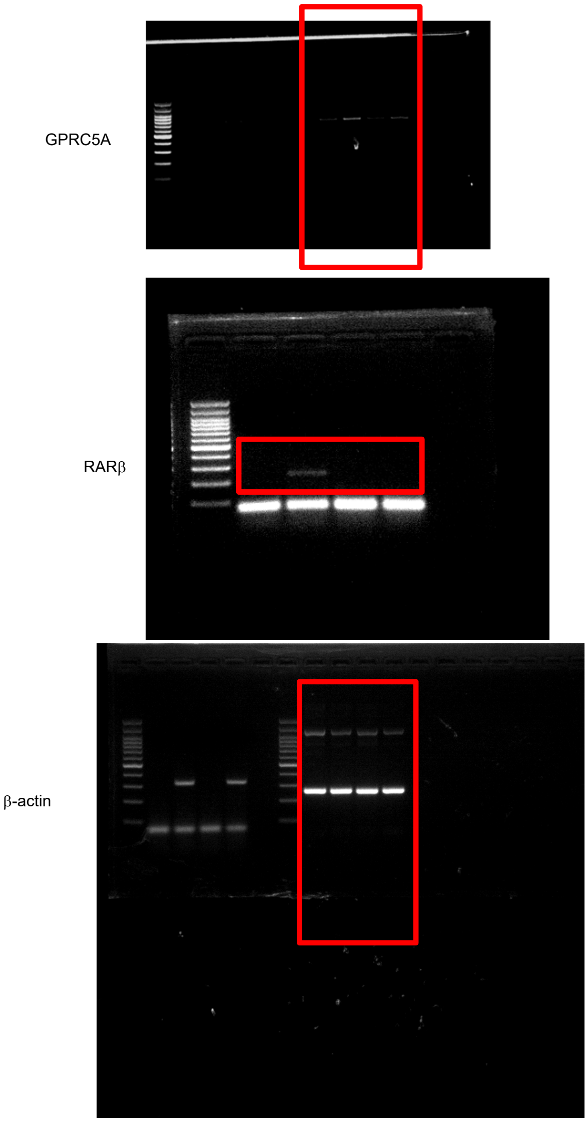

Figure 5B

Old data

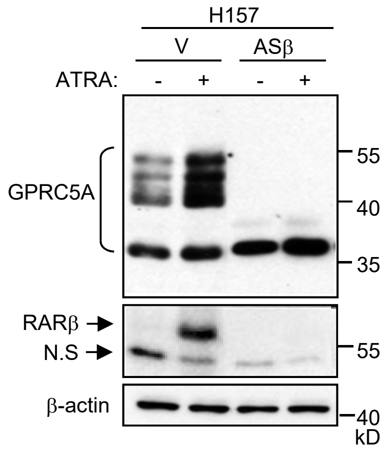

New data

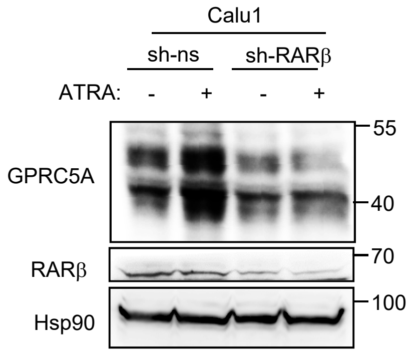

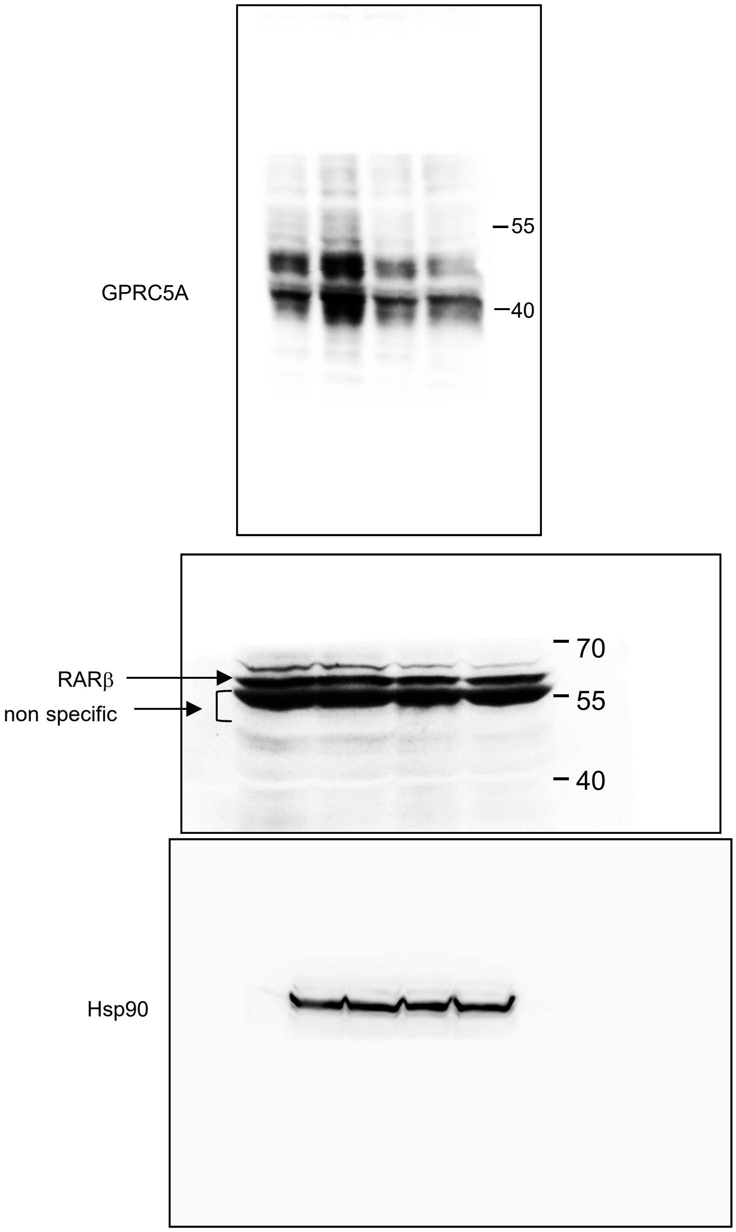

Figure 6C

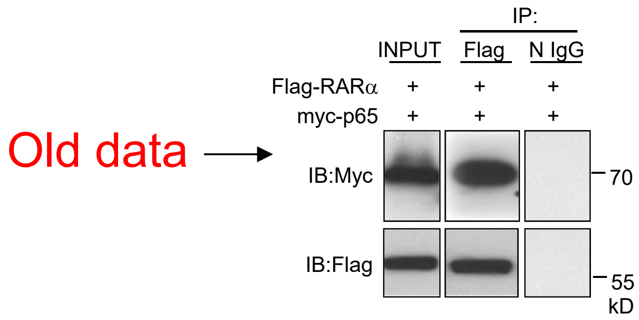

Old data
IP:
INPUT
Flag
N IgG
Flag-RARα
myc-p65
+
IB:Myc
IB:Flag
70
55
kD

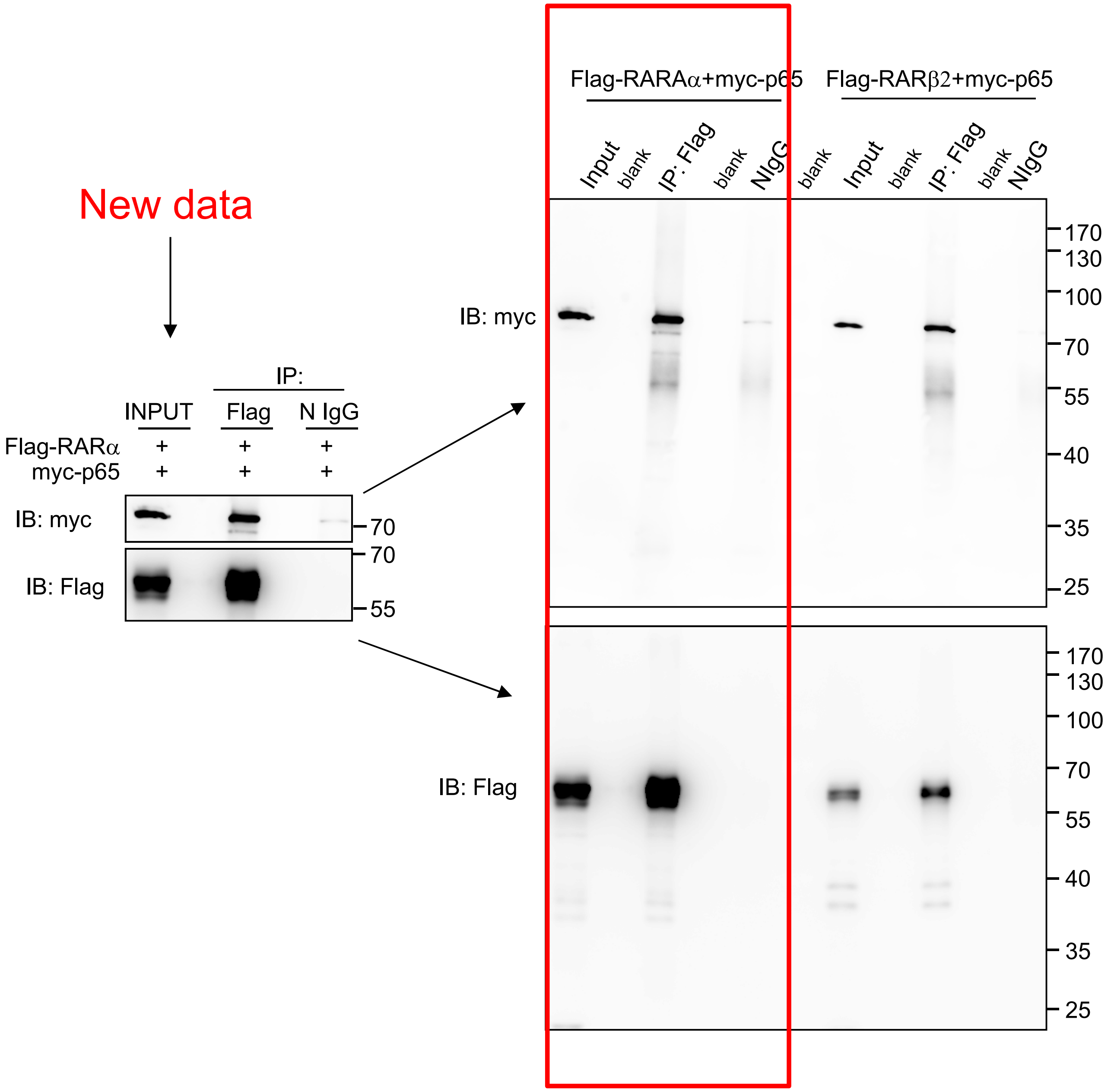

New data
IP:
INPUT
Flag
N IgG
Flag-RARα
myc-p65
+
IB: myc
IB: Flag
70
55
Flag-RARAα+myc-p65
Flag-RARβ2+myc-p65
Input
blank
IP: Flag
NIgG
170
130
100
40
35
25

Figure 6D

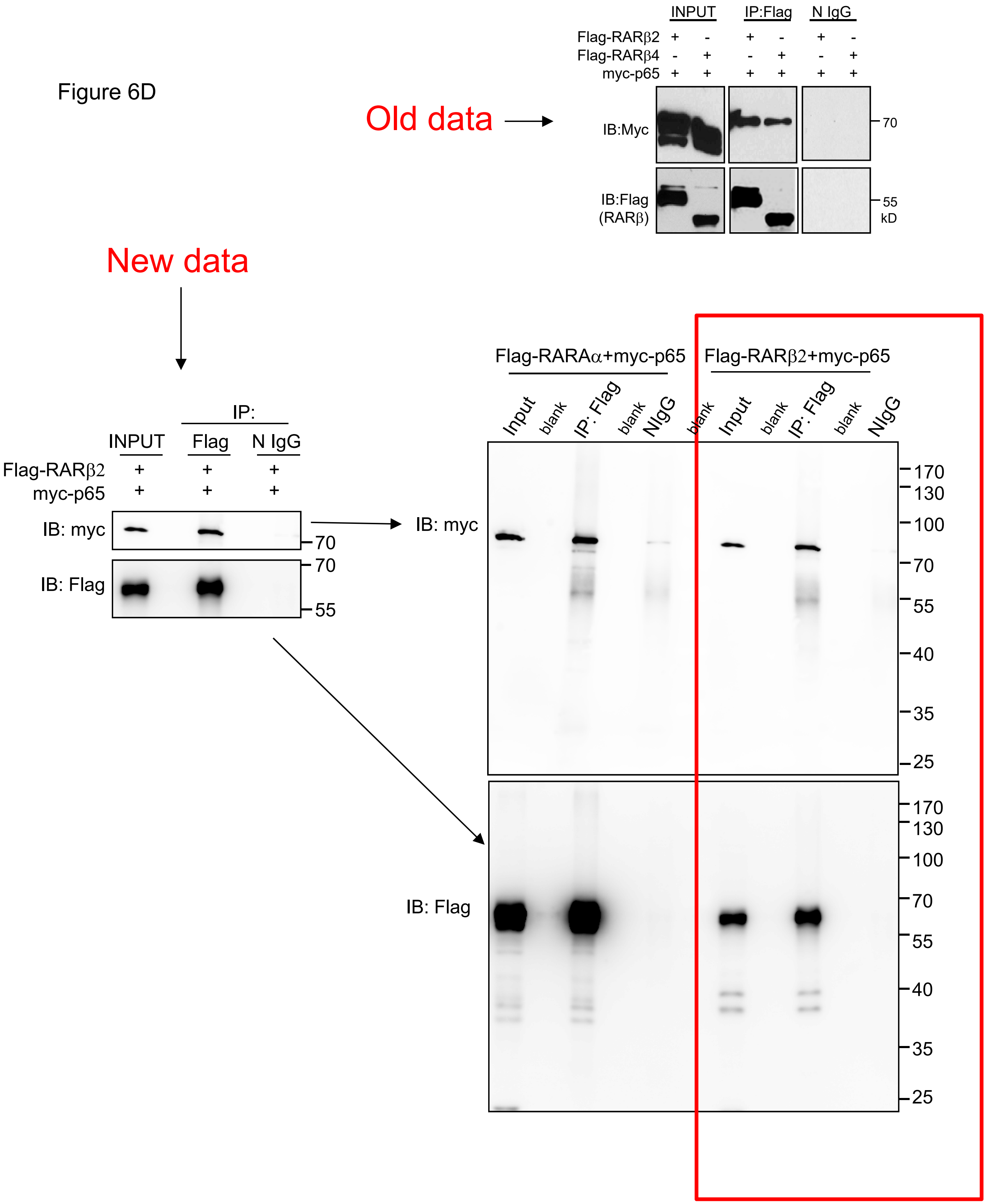
Old data
INPUT
IP:Flag
N IgG
Flag-RARβ2 + - + - + -
Flag-RARβ4 - + - + - +
myc-p65 + + + + + +
IB:Myc
IB:Flag
(RARβ)
70
55
kD
New data
IP:
INPUT
Flag
N IgG
Flag-RARβ2 + + +
myc-p65 + + +
IB: myc
IB: Flag
70
70
55
Flag-RARAα+myc-p65
Flag-RARβ2+myc-p65
Input
blank
IP: Flag
blank
NIgG
blank
Input
blank
IP: Flag
blank
NIgG
IB: myc
IB: Flag
170
130
100
70
55
40
35
25
170
130
100
70
55
40
35
25
